# Virus induced dysbiosis promotes type 1 diabetes onset

**DOI:** 10.3389/fimmu.2023.1096323

**Published:** 2023-01-19

**Authors:** Zachary J. Morse, Rachel L. Simister, Sean A. Crowe, Marc S. Horwitz, Lisa C. Osborne

**Affiliations:** ^1^Department of Microbiology & Immunology, Life Sciences Institute, University of British Columbia, Vancouver, BC, Canada; ^2^Department of Earth, Ocean, and Atmospheric Sciences, University of British Columbia, Vancouver, BC, Canada

**Keywords:** type 1 diabetes, viral-induced autoimmunity, coxsackievirus, microbiome, fecal microbiota transplant (FMT)

## Abstract

Autoimmune disorders are complex diseases of unclear etiology, although evidence suggests that the convergence of genetic susceptibility and environmental factors are critical. In type 1 diabetes (T1D), enterovirus infection and disruption of the intestinal microbiota are two environmental factors that have been independently associated with T1D onset in both humans and animal models. However, the possible interaction between viral infection and the intestinal microbiota remains unknown. Here, we demonstrate that Coxsackievirus B4 (CVB4), an enterovirus that accelerates T1D onset in non-obese diabetic (NOD) mice, induced restructuring of the intestinal microbiome prior to T1D onset. Microbiome restructuring was associated with an eroded mucosal barrier, bacterial translocation to the pancreatic lymph node, and increased circulating and intestinal commensal-reactive antibodies. The CVB4-induced change in community composition was strikingly similar to that of uninfected NOD mice that spontaneously developed diabetes, implying a mutual “diabetogenic” microbiome. Notably, members of the Bifidobacteria and Akkermansia genera emerged as conspicuous members of this diabetogenic microbiome, implicating these taxa, among others, in diabetes onset. Further, fecal microbiome transfer (FMT) of the diabetogenic microbiota from CVB4-infected mice enhanced T1D susceptibility and led to diminished expression of the short chain fatty acid receptor GPR43 and fewer IL-10-expressing regulatory CD4^+^ T cells in the intestine of naïve NOD recipients. These findings support an overlap in known environmental risk factors of T1D, and suggest that microbiome disruption and impaired intestinal homeostasis contribute to CVB-enhanced autoreactivity and T1D.

## Introduction

Type 1 diabetes (T1D) is an autoimmune disease that results from activation of self-reactive leukocytes that destroy the insulin-secreting beta cells in the pancreas, leading to a subsequent loss of blood glucose regulation. Like many autoimmune disorders, T1D incidence has increased globally in recent years (1, 2). Notably, the concordance rate of T1D in monozygotic twins is ~50% (3), suggesting that genetics and heritability only partially account for the likelihood of developing the disease. Environmental factors, including infection history and features of the intestinal microbiome, cause extensive variation in the immune system (4) and have been prominently implicated in T1D pathogenesis (5–7).

Both clinical and epidemiological studies show that infections of Coxsackievirus B (CVB) and other enteric viruses play a role in precipitating autoimmunity in T1D-susceptible individuals (8–12). CVB is an enterovirus with a single-stranded RNA genome that is typically transferred *via* the fecal-oral route in humans. Furthermore, enteric infection and persistent gastrointestinal (GI) inflammation precedes T1D onset in humans, signifying a link between intestinal immunity and altered islet-reactivity (9, 13, 14). Clinical and epidemiological findings show that CVB infection is a risk factor for T1D, and we have previously found that CVB4 infection is sufficient to break immune tolerance in non-obese diabetic (NOD) mice and accelerate the onset of autoimmune diabetes by promoting bystander immune activation and innate antiviral responses involving type I interferon production (15–18).

Dysbiosis, defined here as a substantial alteration of the intestinal microbial community and/or its functional capacity in relation to the host, has been shown to contribute to the pathogenesis of several inflammatory disorders (19). In T1D, changes in microbial community composition, metabolite production, and the intestinal virome have been associated with disease susceptibility and immune regulation in both humans and rodent models (20–24). Certain T1D-susceptibility alleles for immune-related loci may promote development of autoimmunity *via* modulation of the gut microbiome, particularly early in life (21, 25). Accordingly, disruption in the intestinal microbiome by infection, diet, antibiotic use, or other factors, plays a considerable role in precipitating autoimmune diabetes (26, 27).

Immune homeostasis in the gastrointestinal (GI) tract and associated lymphoid tissue (GALT) can modify T1D clinical outcomes, contributing to either protection from or pathogenesis of T1D. Protective mechanisms include the migration of IL-10-expressing type 1 regulatory T cells from the GALT to the pancreas to induce tolerance (28), and the production of microbial metabolic products such as short-chain fatty acids (SCFA), including acetate and butyrate, that limit the frequency of autoreactive T cells in T1D (22). Specifically related to virus-microbiome influences on T1D, murine norovirus infection protects against T1D in NOD mice *via* modification of the intestinal microbiota and immune landscape (29). In contrast, intestinal inflammation can promote the presentation of pancreatic self-antigen and activation of autoreactive immune cells, particularly within the pancreatic lymph nodes (PLN) that drain GI and pancreatic tissues (30, 31). Collectively, these reports suggest the existence of a gut-pancreas axis that links the intestinal microbiome and inflammatory events with immune homeostasis and the autoreactive potential of the pancreatic islets (32).

The importance of both the intestinal microbiome and viral infection to T1D pathogenesis is well established in both humans and *in vivo* mouse models, but the interaction between these environmental risk factors has not been defined. Given previous data demonstrating that CVB4 infection accelerates diabetes onset, that viral infections can cause intestinal dysbiosis (29, 33–35), and that dysbiosis influences T1D pathogenesis (27, 36–38), we hypothesized that CVB4 infection may modify the intestinal microbiome in a way that enhances diabetogenic potential. Here, we demonstrate that CVB4 infection of NOD mice elicits reproducible changes in the microbiome that precede T1D onset. Infection also resulted in eroded intestinal mucosa, impaired epithelial barrier integrity, and bacterial translocation that was associated with enhanced humoral recognition of commensal antigen. Further, fecal microbiome transfer (FMT) of pre-diabetogenic feces from CVB4-infected donors was sufficient to enhance T1D susceptibility of virus-naive recipients. Collectively, these data demonstrate that enteroviral infection can shift the intestinal microbial community composition and structure in a manner that promotes disease susceptibility and as such creates a potential therapeutic target and/or biomarker for earlier detection of T1D susceptibility and subsequent disease prevention.

## Results

### Microbial dysbiosis is a consequence of coxsackie virus B4 infection

**Figure 1:**
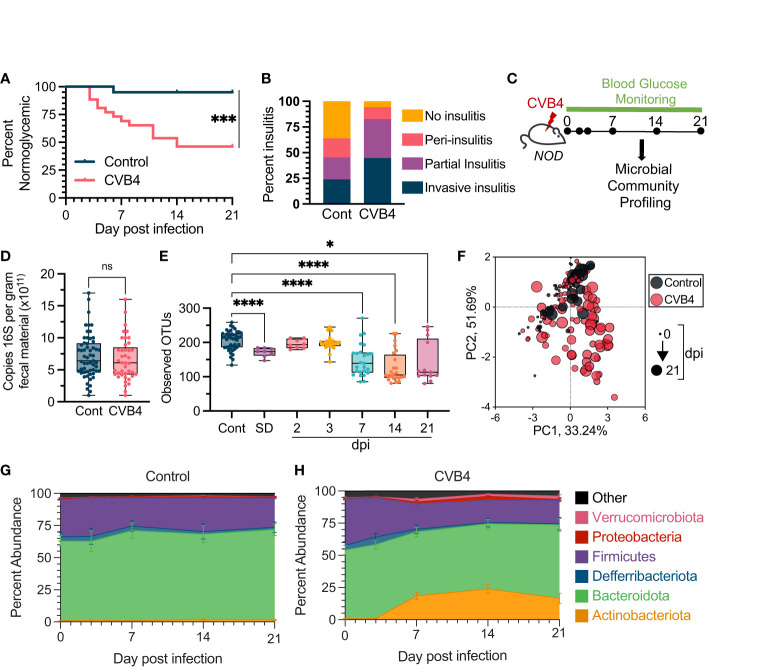
Microbial Dysbiosis is a Consequence of Coxsackievirus B4 (CVB4) Infection **(A)** Diabetes incidence in uninfected control (n = 20) vs. CVB4-infected (n = 26) mice as determined by blood glucose measurement. Data are combined from 3 independent experiments and analyzed using a log-rank (Mantel-Cox) test. **(B)** Degree of pancreatic insulitis as (at day 21 pi) determined by histology (n = 5-6 per group). **(C)** Timeline of microbial community profiling. Fecal samples were collected at days 0, 2, 3, 7, 14, and 21 days pi. **(D)** Copies of 168 genes per gram of fecal material as measured by qPCR (CVB4 = days 2-21 pi). P value calculated using paired Welch's t test (two-tailed). **(E)** a-diversity (Observed number of OTUs) in bacterial communities following CVB4 infection. Control group includes baseline samples from all mice in the experiment on day 0 prior to mock or CVB4 infection. P values were calculated using Welch's ANOVA with Dunnet's T3 multiple comparisons test. **(F)** Principal component analysis (PCoA) of microbial community compositions. Longitudinal changes in the microbiome of **(G)** uninfected control (n=20) and **(H)** CVB4-infected mice (n=26) at the phylum level throughout sampling (Days 0, 3, 7, 14, and 21 pi). Data include replicates from at least three independent experiments. Results in D and E are represented as mean ± range, G and H are mean ± standard error. *P≤0.05 was considered statistically significant; ***P≤0.001; ****P≤ 0.0001, ns, not significant.

To interrogate the interplay of enteric viral infection and the microbiome in the context of T1D pathogenesis, we infected NOD mice with CVB4 and monitored onset of diabetes by blood glucose measurements taken every 2-3 days. Consistent with previous studies from our group (15, 17), roughly half (53.8%) of the CVB4-infected mice became diabetic within 2 weeks, while only 5% of their mock-infected (control) counterparts spontaneously developed diabetes over the same period (**Figure 1A**). Virus-induced acceleration of T1D onset was also sex-independent and affected both female and male NOD mice equally (**Figures S1A, B**). Expectedly, the elevated incidence of diabetes in infected mice also resulted in increased insulitis of the pancreatic islets (**Figures 1B**; **S1C**).

To directly assess the impact of CVB4 infection on the intestinal microbial community, fecal pellets were longitudinally collected and analyzed from mock-infected naïve (control) and infected mice at days 0, 2, 3, 7, 14 and 21 post-infection (pi) (**Figure 1C**). All mice, independent of glycemia, were included in subsequent microbiome analyses. While CVB4 infection did not affect the overall quantity of fecal bacterial 16S rRNA gene copies (**Figure 1D**), there were substantial infection-induced changes to microbial community composition and structure over time (**Figures 1E-H**). By day 7 pi, CVB4-infected mice demonstrated a significant loss of α-diversity *via* multiple measures of the richness and/or evenness of the microbial community in a given sample of the fecal microbiome that was sustained through to the end of our experiments (21 days pi) (**Figures 1E**; **S2**). Notably, mice that developed diabetes spontaneously (SD) also demonstrated reduced α-diversity compared to normoglycemic control mice (**Figures 1E**; **S2**). Further, while the β-diversity (similarity/dissimilarity of bacterial communities between samples) of the fecal microbial community from uninfected, non-diabetic was stable throughout the 21-day monitoring period, there was a significant shift from baseline in the fecal microbiome of CVB4-infected mice over the same time period (**Figure 1F**; **Table S1**). At the phylum level, baseline communities were comprised mostly of members of Bacteroidota and Firmicutes, which was sustained over the course of sampling in control mice (**Figure 1G**). In contrast, communities from infected mice underwent rapid reorganization within the first week post-CVB4 infection characterized by increased relative abundance of Actinobacteria and Verrucmicrobiota phyla (**Figures 1F, H**). Luminal contents taken from the small intestine and proximal colon also exhibited a loss of bacterial α-diversity and a dysbiotic profile (**Figure S3**) suggesting that fecal samples appropriately reflect changes within the GI environment.

### Bacterial indicators of CVB4-induced dysbiosis associated with T1D

**Figure 2:**
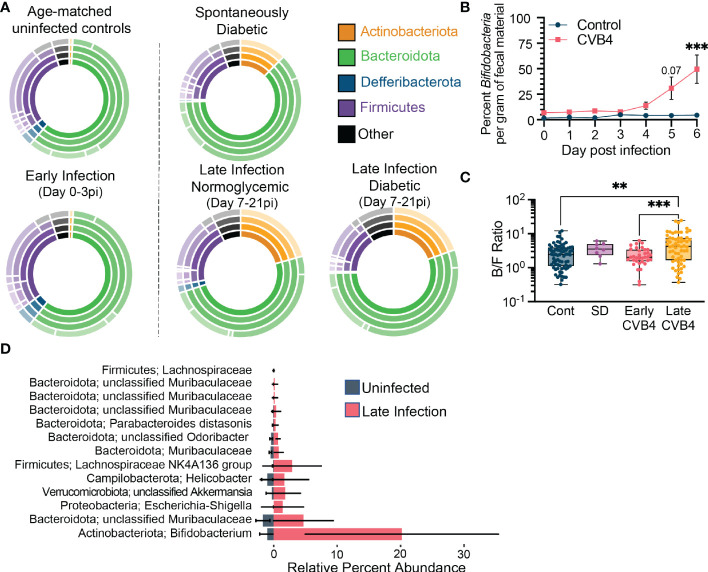
Bacterial Indicators of CVB4-induced Dysbiosis Associated with T1D **(A)** The relative abundance of bacterial taxa within experimental groups. Inner rings indicate composition on a phylum level and each subsequent ring represents a lower taxonomic level (class, order, family). Uninfected (normoglycemic, days 0-21 pi, n = 72); Early infection, days 0-3 pi (n = 34); Late infection - Normoglycemic, days 7-21 pi with blood sugar <16.2 mg/dL (n = 38); Late infection - Diabetic, days 7-21 pi with blood sugar >16.2 mg/dL (n = 24); Spontaneous diabetic, no infection with blood sugar >16.2 mg/dL (n = 8). **(B)** Copies of Bifidobacteria genes per gram of fecal material over the first 6 days of infection as measured by qPCR. (Control n = 3, CVB4 n = 6). Data are from one experiment and analyzed by Two-Way ANOVA using Šidak's multiple comparison test. **(C)** Ratio of Bacteroidota to Firmicutes abundance in fecal pellets of control, spontaneously diabetic (SD) and CVB4-infected mice at days 0-3 pi (early infection) and 7-21 pi (late infection). Data are from 3 independent experiments and represented as mean ± range. P values were calculated using Welch's ANOVA with Dunnet's T3 multiple comparisons test. **(D)** Lefse analysis displaying indicator bacterial species associated with uninfected control samples vs. late infection timepoints (Day 7-21pi) (Uninfected n = 72, Late Infection n = 62 samples). All data are representative of at least three independent experiments unless stated otherwise. **P≤0.01 was considered statistically significant; ***P≤0.001

Mouse fecal microbial communities were grouped by infection status, time-point, and glycemia (**Figure 2A**). In the first 3 days of CVB4 infection (“Early Infection”, 0-3 dpi) there was no appreciable effect on the fecal microbiome at the phylum level (**Figure 2A**; **Table S1**, **S2**). However, between days 3-7 pi there was a substantial alteration in microbial community composition that was sustained through the 21-day monitoring period (**Figure S4**; **Table S1**). Notably, there was a substantial increase in the relative abundance of the phyla Actinobacteria and Verrucomicrobiota and a contraction in the relative abundance of Firmicutes in fecal samples collected at “Late Infection” time points (7-21 dpi), regardless of glycemia (**Figures 2A**; **S4**). These CVB4-induced changes were remarkably similar to the fecal microbiome composition of the few uninfected mice that developed spontaneous diabetes (**Figure 2A**), suggesting that CVB4 infection may accelerate the acquisition of a diabetogenic microbiome. In mice that did develop diabetes, either CVB4-induced or spontaneous, these changes were apparent prior to hyperglycemia (**Figure S5**). Overall, these data demonstrate that the accelerated diabetes onset caused by CVB4 infection is preceded by a robust and sustained shift in the intestinal microbiome.

To evaluate more nuanced aspects of microbial community compositions and their dynamics, we tracked the abundances of bacterial genera and species responsible for the dysbiosis associated with infection and diabetes susceptibility. In naïve mice and in the Early Infection timepoints (0-3 days pi), the fecal microbial community featured relatively abundant Bacteriodota and Firmicutes, primarily composed of three genera: an unclassified member of the *Muribaculaceae* family, *Alistipes*, and an unclassified member of the *Lachnospiraceae* family (**Figure S6**). CVB4 infection elicited an increase in *Bifidobacteria* (within the Actinobacteriota phylum) and *Akkermansia* (within the Verrucomicrobiota phylum) genera that became prominent in Late Infection stages (days 7-21 pi). Similar elevations in Bifidobacteria and Akkermansia were noted in mice that developed spontaneous diabetes (**Figure S6**). Using *Bifidobacteria* abundance as a metric, we identified signs of infection-induced dysbiosis as early as day 5 pi (**Figure 2B**). Compensatory loss of strains within the Firmicutes phylum (**Figure S4**) resulted in an increased Bacteroidota to Firmicutes ratio (**Figure 2C**) in infected mice.

LEfSe analyses identified a number of additional indicator taxa that have elevated abundances following infection (**Figure 2D**), as well as several taxa associated with uninfected, early infection, and late infection experimental groups (**Tables S3-5**). In addition to the *Bifidobacteria* and *Akkermansia*, analyses also associated an unclassified species of the *Lachnospiraceae*, *Helicobacter*, and a number of unclassified species of *Muribaculaceae* with CVB4 infection (**Figure 2D**). However, the similarity between CVB4 infected mice that develop hyperglycemia and those that remain normoglycemic suggests that much like other risk factors associated with T1D (e.g. autoantibodies, autoreactive T cells, insulitis, dysbiosis, MHC I and interferon-stimulated gene hyperexpression in the pancreas, etc.), an altered microbiome may increase T1D susceptibility without independently dictating disease incidence.

### Altered intestinal physiology and bacterial translocation in CVB4-infected NOD mice

**Figure 3:**
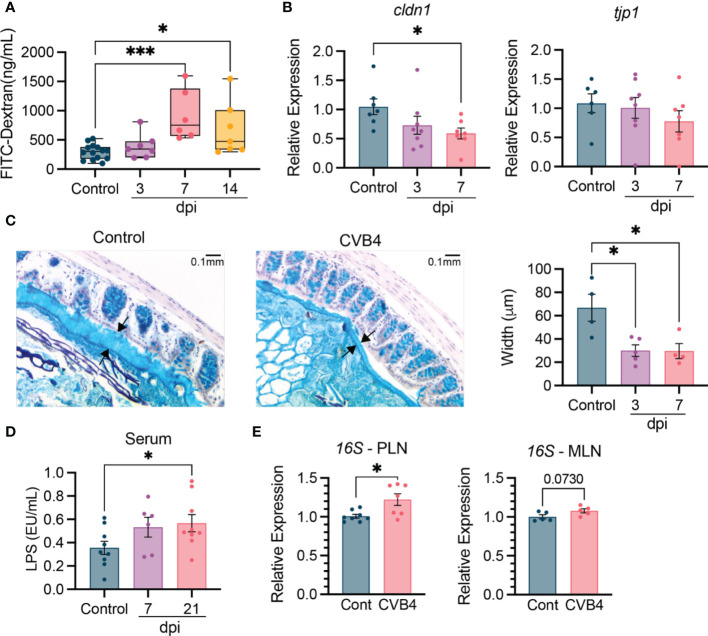
Altered intestinal physiology and bacterial translocation in CVB4-infected NOD mice NOD mice (11-12 weeks) were infected with 400 pfu CVB4 intra-peritoneally. **(A)** Intestinal permeability based on detection of orally gavaged FITC-Dextran (4 kD) in the serum. Data are combined from four independent experiments. **(B)** Gene expression levels of tight junction-related proteins claudin 1 and tight junction protein 1 in the proximal colon of naïve and CVB4-infected mice. Target gene expression was normalized to GAPDH and expressed relative to uninfected control mice. Data are combined from two independent experiments. **(C)** Representative images of Alcian Blue stained mucus (between arrows) from control and CVB4-infected mice (left) and quantification of mucus width (right). **(D)** LPS endotoxin levels in the serum. Samples collected from two independent experiments were run on single assay. **(E)** Detection of 16S rRNA in the MLN and PLN of control and CVB4-infected mice (day 7 pi) as determined by qPCR. Intestinal permeability is represented as median with range. All other results are expressed as the mean ± SEM. P values in A-D were calculated using Welch's ANOVA with Dunnet's T3 multiple comparisons test and E was calculated using paired Welch's r test (two-tailed). *P<0.05 was considered statistically significant; *** P≤ 0.001.

Given that CVB4 accelerated T1D onset is associated with altered microbial community composition, we assessed whether intestinal physiology was also affected. Notably, deliberate disruption of intestinal integrity is sufficient to activate islet-specific autoreactive T cells within the gut mucosa and accelerate diabetes onset in NOD mice (39). By day 7 pi, a time point that coincides with established microbiome shifts in diabetic mice, serum levels of FITC-dextran were elevated by ~2-fold compared to uninfected, non-diabetic controls, indicating reduced intestinal barrier integrity that was maintained until at least day 14 pi (**Figure 3A**). These changes were associated with reduced colonic expression of the gene encoding tight junction-associated protein claudin-1 (*cldn1*) and a slight decrease in tight junction protein-1 (*tjp1*) gene expression (**Figure 3B**). A layer of mucus largely comprised of glycoproteins produced by intestinal goblet cells acts as a physical barrier to maintain separation of commensal bacteria and the host epithelium. Notably, CVB4 infection induced a rapid thinning of the colonic mucosal barrier (**Figure 3C**) that coincided with altered expression of mucin and antimicrobial peptide (amp) genes within the ileum and proximal colon (**Figures S7A, B**), indicating broad effects on intestinal epithelial cell function within the first week of CVB4 infection of NOD mice that coincide with changes in the intestinal microbiome.

The CVB4-induced changes in intestinal physiology suggested that colonizing microbes may be more likely to translocate out of the gut and into other tissues. Supporting this hypothesis, we found an increased abundance of bacterial lipopolysaccharide (LPS) detected in the blood as early as day 7 pi that was maintained through day 21 pi, suggesting persistent changes to overall intestinal physiology following CVB4 infection (**Figure 3D**). Moreover, elevated levels of bacterial 16S rRNA were detected in isolated PLN at day 7 post-CVB4 infection (**Figure 3E**). 16S rRNA was also slightly elevated, yet not significantly, in the mesenteric (MLN) lymph nodes, suggesting the PLN may be preferentially affected (**Figure 3E**). Collectively, these data indicate that CVB4 infection contributes to systemic and local inflammation through translocation of bacterial antigen.

### CVB4 modifies host responses to commensal bacteria

**Figure 4:**
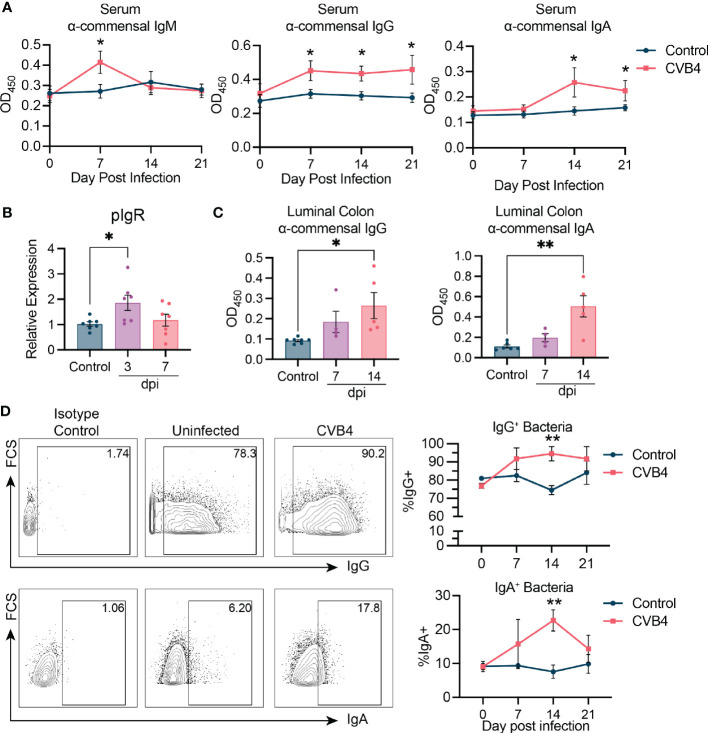
CVB4 modifies host responses to commensal bacteria **(A)** Abundance of bacterial antigen-specific IgM, IgG, or IgA in the serum as measured by ELISA (n = 5-6 mice per group). Each sample was run in duplicate and averaged. **(B)** Relative expression of polymeric Ig receptor in the small intestine as determined by RT-qPCR. **(C)** Colonic luminal IgA and IgG reactivity to bacterial antigen as measured by ELISA. **(D)** Flow cytometry of bacteria from the feces of infected mice indicating proportion of microbes coated with either IgG (top) or IgA (bottom) antibodies. Representative flow plots showing gating of IgG and IgA bound microbes (left), and quantification on the right (n = 4 mice per group). Results are expressed as the mean ± SEM. P values in A and D are calculated by Two-Way ANOVA with Sidák's multiple comparisons test while those in B and C were calculated via Welch's ANOVA with Dunnet's T3 multiple comparisons test. *P<0.05 was considered statistically significant; **P≤0.01.

Antibody responses to commensal bacteria are typically triggered following introduction of novel bacteria or by new exposure of microbial antigens to immune cells (40). Notably, dysregulation of anti-commensal antibodies has been associated with T1D autoimmunity in both humans and mice (41, 42). To test whether impaired barrier integrity, the restructured microbiome, and bacterial translocation in CVB4-infected mice impacted humoral immunity to commensal bacteria, we assayed the presence of commensal-reactive circulating IgM, IgA, and IgG at days 7, 14, and 21, pi. Following a transient increase in commensal-specific IgM at day 7 pi, IgG was elevated by day 7 pi until day 21 pi, while IgA levels peaked at day 14 pi and were maintained through day 21 pi (**Figure 4A**). Consistent with this, expression of the polymeric Ig receptor (pIgR) increased following infection, signifying accelerated trafficking of immunoglobulins across the epithelial barrier (**Figure 4B**). This was reflected in detection of increased commensal-reactive IgA and IgG in the colonic lumen (**Figure 4C**) and elevated binding of luminal antibodies to microbes isolated from feces (**Figure 4D**), indicating that the increased luminal trafficking of immunoglobulins impacts the binding and neutralization of bacteria within the colon of CVB4-infected mice. Ultimately, the elevated antibody responses to intestinal microbes in response to CVB4 infection may indicate a compensatory reaction by the host to stabilize or limit dysbiosis.

### The CVB4-modified microbiome is diabetogenic

**Figure 5:**
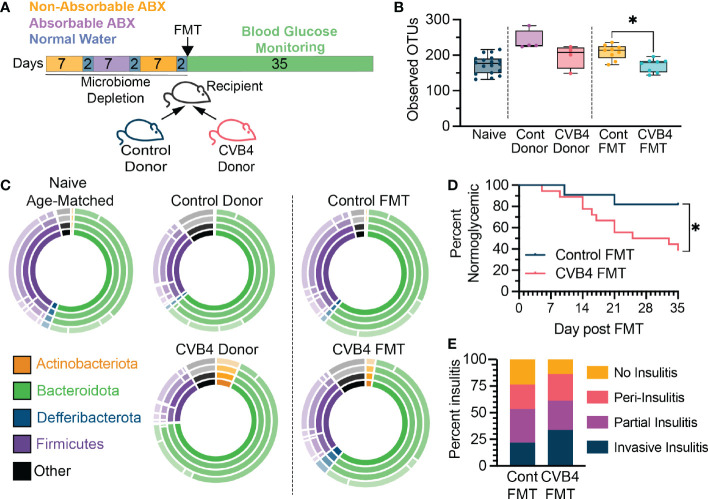
The CVB4-modified microbiome is diabetogenic **(A)** Experimental design for antibiotic depletion of recipients followed by FMT from donor mice. **(B)** Microbial a-diversity (Observed OTUs) in reconstituted FMT recipients. P values calculated via Welch's ANOVA with Dunnet's T3 multiple comparisons test. **(C)** The relative abundance of bacterial taxa within FMT recipient mice at day 35 post-FMT. Inner ring represents phyla and concentric rings moving out represent lower taxonomic levels (class, order, family) **(D)** Diabetes incidence in mice receiving FMT from either control (n=11) or a previously infected donor (n = 18) collected at day 14 pi. P value calculated by log-rank (Mantel-Cox) test. **(E)** Insulitis levels in mice receiving FMTs at 5 weeks post FMT. All mice in **(D)** were included in microbiome analyses and insulitis measurements. All data in this figure is representative of four independent experiments. A total of four donor pairs (control vs CVB4-infected) were tested. statistically significant. *P<0.05 was considered statistically significant.

To assess whether the CVB4-induced dysbiotic microbiome contributes to the acceleration of autoimmune diabetes, we first used a course of broad-spectrum antibiotics to deplete the microbial biomass of 6-week-old NOD mice (**Figure 5A**) before infecting them with CVB4. We found that a majority (71%) of microbiome-depleted mice succumbed to CVB4 infection within 7-10 days (**Figure S8**), confounding assessment of whether the microbiome is necessary for virus-induced autoimmune diabetes acceleration. Therefore, we turned to fecal microbiome transfer (FMT).

To directly evaluate the diabetogenic potential of a CVB4-modified microbiome, microbiome-depleted naïve mice received an FMT from either naïve (control FMT) or previously CVB4-infected (CVB4 FMT) donors and blood glucose was monitored over the next 5 weeks (**Figure 5A**). Consistent with previous reports indicating that CVB4 is not transmitted *via* the fecal-oral route (43) there was no detectable virus in the donor samples or recipient mice, and no histological evidence of viral infection in FMT recipients (data not shown). Notably, the fecal microbiome of FMT recipients was similar to the donor material both in terms of reduced α-diversity in CVB4 FMT compared to controls, as well as overall bacterial community composition (**Figures 5B, C**). Remarkably, female recipients of a CVB4 FMT were more susceptible to autoimmune diabetes (61.2% hyperglycemic at 5 weeks post-FMT) as compared to sex-matched recipients of a control FMT (18.2% hyperglycemic at 5 weeks post-FMT) (**Figure 5D**). Accordingly, female recipients of the CVB4 FMT also displayed greater pancreatic insulitis (**Figure 5E**). However, none of the male mice receiving FMT from naïve or CVB4-infected donors became diabetic in the 5 weeks following FMT (data not shown). Thus, these data indicate that although a CVB4-modified microbiome is not sufficient to break immune tolerance characteristic of male NOD mice, it does significantly alter host responses in females that are more susceptible to spontaneous T1D onset. Overall, the dysbiosis that results from and persists following enteric virus infection can skew host homeostasis to promote the development of diabetes autoimmunity even in the absence of viral infection.

### FMT modifies intestinal immunity

**Figure 6:**
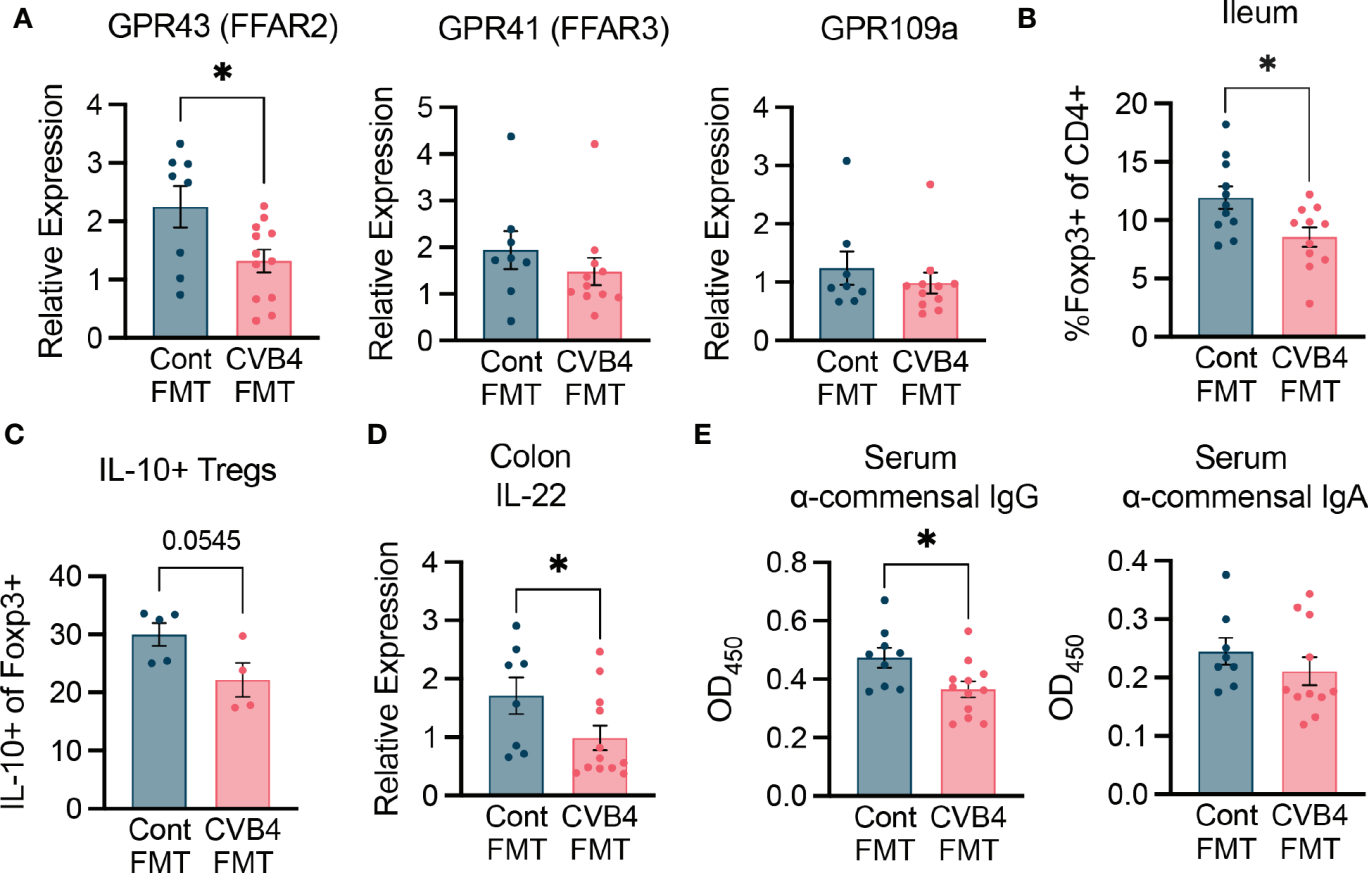
FMT modifies intestinal immunity **(A)** Expression of free fatty acid receptors in the proximal colon of FMT recipient mice. **(B)** Abundance of CD4 Foxp3+ regulatory T cells in the ileum of FMT mice. **(C)** Expression of IL-10 in the intestinal Tregs. **(D)** Expression of IL-22 in the proximal colon measured by RT-qPCR. Results were normalized to GAPDH and expressed as relative expression compared to age-matched naive controls. **(E)** Abundance of anti-commensal antibodies in the serum of FMT recipient mice as measured by ELISA. All samples were analyzed at day 35 post-FMT. All p values were calculated using paired Welch's test (two-tailed) from at least 2 independent experiments. *P<0.05 was considered statistically significant.

To determine how the dysbiotic microbiome of the CVB4 FMT impacts immune homeostasis and contributes to diabetes susceptibility, we characterized responses within the intestine of female FMT recipients. Short-chain fatty acids (SCFAs) produced by commensal microbes through fermentation of dietary fiber activate free-fatty acid receptors including GPR43, GPR41, and GPR109a to promote regulatory immune responses. The most abundantly expressed of these receptors, GPR43, had reduced expression in the CVB4 FMT mice compared to that of the control FMT recipients (**Figure 6A**) suggesting reduced SCFA signaling. Since signaling through GPR43 and other free fatty acid receptors is known to affect regulatory immune responses (44, 45), we quantified and assessed the function of T1D-protective regulatory T cells (Tregs) within the lamina propria of the small intestine. Relative to controls, mice receiving an FMT of the dysbiotic microbiome had a lower abundance of anti-inflammatory Foxp3^+^ regulatory CD4^+^ T cells (Tregs) and reduced capacity for IL-10 cytokine production (**Figures 6B, C**).

Further, gene expression of the cytokine IL-22, which is a crucial regulator of epithelial homeostasis and microbial defense, was lower in the proximal colon of CVB4 FMT mice relative to control FMT recipients (**Figure 6D**). While innate immune signaling through toll-like receptors in colonic tissue was not significantly altered (**Figure S9**), CVB4 FMT recipient mice had lower systemic anti-commensal IgG (but not IgA) responses (**Figure 6E**) than the controls, indicating that dysbiosis may lead to a reduced capacity for the host to neutralize commensal antigens and maintain homeostasis. Collectively, these results suggest microbiome dysbiosis resulting from CVB4 infection is capable of modifying the intestinal landscape in a way that is sufficient to promote diabetes autoimmunity.

## Discussion

Herein, we examined the combined effects of two environmental factors, enterovirus infection and dysbiosis, that have been independently implicated in T1D pathogenesis. In this study, we show that CVB4 infection is capable of driving microbiome dysbiosis, disrupting intestinal physiology, promoting bacterial translocation, and altering host responses to commensal bacteria to ultimately promote the onset of autoimmune diabetes in NOD mice.

While microbiome studies display a good deal of heterogeneity depending on populations studied and conditions of the subjects, there are some consistent results between people with T1D autoimmunity and mice infected with CVB4. These include a loss of α-diversity within bacterial communities, increased abundance within the Bacteroidota phylum relative to a decrease in Firmicutes, and alteration of the bacterial metabolome (22, 23, 42, 46). Our data are consistent with independent clinical observations that childhood CVB infection is a risk factor for T1D (8, 10, 20, 47), and that dysbiosis can be detected in humans prior to T1D development (27, 38), and indicate that CVB infection may drive disease-associated microbiome restructuring.

Increased gut permeability is associated with a number of autoimmune diseases, including T1D, multiple sclerosis, and systemic lupus erythematosus (48). This permeability has been identified prior to diabetes development in clinical settings as well as mouse models, suggesting that it may serve as a promoter or initiator of autoreactivity. Individuals with islet autoimmunity often have deficiencies in intestinal integrity along with mild enteropathy (14, 49). In fact, inducing a “leaky gut” is sufficient to trigger development of islet-reactive T cells in BDC2.5 transgenic NOD mice and bacterial translocation has been found to precede diabetes onset and act as a contributor to autoreactivity in NOD mice (39, 50, 51). Specifically, increased gut permeability can stimulate islet-reactive T cells in the GI environment to migrate to the pancreas and participate in destruction of the insulin-secreting beta cells (39). Since CVB4 infection is able to induce GI permeability, it represents a potential secondary mechanism that can contribute to autoreactivity.

Infection with CVB4 led to a loss of intestinal integrity and bacterial translocation to the PLN. The intestinal microbiota of CVB4-infected NOD mice had elevated relative abundances of *Akkermansia muciniphilia*, which is a known mucin-degrading bacterium due to its ability to catabolize glycans. Overgrowth by members of the *Akkermansia* genus is commonly associated with degradation of the epithelial mucosal barrier, implicating these bacteria in the diminished intestinal mucus barrier we observed following CVB4 infection. Increased gut permeability leads to elevated risk of bacterial exposure to immune cells residing in both the mucosal and peripheral environments with potential for subsequent promotion of inflammation. Due to intestinal alteration following CVB4 infection, bacteria are able to escape the GI tract and enter circulation and PLN. Bacterial translocation promotes diabetes through several mechanisms, including bacterial antigen presentation to autoreactive T cells (39), enhanced inflammation (52), molecular mimicry (53, 54), and direct damage to the islet microenvironment (55). We thus propose that increased bacterial presence systemically and within the local pancreatic environment following infection may increase activation of islet-specific T and B cells that lead to autoimmune destruction of insulin-producing pancreatic beta cells.

Infection with CVB4 increased production of anti-commensal IgG and IgA antibodies, which typically help maintain GI homeostasis by controlling bacterial colonization and spatial distribution, particularly within mucosal environments (56). Differences in anti-commensal responses may play a role in diabetes development since they have been observed in individuals at greater risk of developing T1D due to presence of autoimmune risk alleles (57). Furthermore, these humoral responses can be specific to certain species of bacteria. For example, variance has been observed in the prevalence of antibodies against different strains of *Bifidobacteria* in young children who went on to develop islet autoimmunity and T1D later in life (58). While further work is needed, this indicates a role for the expanded population of *Bifidobacteria* in CVB4-infected NOD mice. Collectively, these data suggest that reactivity to certain commensal microbes may represent a risk factor for T1D development and that microbial community compositions and/or reactivity to members of these communities may provide diagnostic biomarkers.

The intestinal microbiome can be detrimental to immune homeostasis, since strain-specific bacteria can skew T cell polarization into either inflammatory or regulatory pathways and thus contribute to autoimmunity (59, 60). Accordingly, dysbiosis caused by CVB4 infection in mice may alter the antigenic profile of the bacterial milieu and enhance inflammation and/or restrain microbiota-induced tolerization (61). To uncouple antiviral immunity from the immune mechanisms elicited by the resulting dysbiosis, we used an FMT approach. Although CVB4-induced diabetes is sex-independent, only female NOD mice exhibit enhanced susceptibility to the diabetogenic microbiome, suggesting that FMT alone is not sufficient to recapitulate the viral-induced loss of immunological tolerance in male recipients. However, female NOD mice that are susceptible to spontaneous diabetes revealed a role for the CVB4-modified microbiome in T1D pathogenesis that may be due to altered communication with resident immune cells and impaired regulatory responses within the GI environment. Whether there are functional differences in the microbiome of CVB4-infected mice that do or do not go on to develop T1D remains unknown.

Lower GPR43 expression in the diabetogenic microbiome recipient mice indicates a reduction in SCFA signalling, suggesting the dysbiotic microbiome that develops following CVB4 infection may result in altered SCFA production or bioavailability. Production of SCFAs through fermentation of dietary fibers is one of the most important ways commensal microbes communicate with host cells, and the fecal microbiome of people with T1D are characterized by diminished representation of genes associated with fiber fermentation and SCFA biosynthesis (27). SCFAs are able to regulate production of IL-22 within the GI environment as well as binding to host receptors including free-fatty acid receptors (GPR43, GPR41, GPR109a) responsible for cellular gene transcription and cell metabolism (62, 63). SCFAs like butyrate and propionate are known to promote the production, differentiation, and function of intestinal regulatory T cells (22, 44, 64) and thus may contribute to the reduced regulatory responses we observed in the GALT of CVB4 FMT mice.

Collectively, these data advance our understanding of virus-induced autoimmunity. We have shown that enteric viral infection can disrupt both the microbiome and GI-related pathology, thereby promoting the onset of diabetes. This reveals that dysbiosis can exist as a vestige of past infection with potential to affect future health and disease predisposition. Ultimately, it is necessary to determine how infectious pathogens affect the human intestinal microbiome and physiology and identify the associated effects on susceptibility to autoimmunity. As the prevalence of T1D continues to rise in North America and across the globe we need to determine how different environmental factors affect the host individually, or in concert. Further, increased understanding of how the host responds to shifts in the gut microbiome can help guide development both of new therapeutic interventions and novel biomarkers of disease susceptibility.

## Materials and methods

### Experimental design

The objective of this study was to determine how microbial and viral environmental factors engage in cross-communication with host immunity to precipitate type 1 diabetes in genetically susceptible NOD mice. The T1D-associated virus, CVB4, was assessed for its ability to induce intestinal dysbiosis in NOD mice by performing microbial community profiling using 16S gene sequencing, and the functional effects of CVB4-induced dysbiosis on T1D onset and immune dysregulation were assessed using fecal microbial transplants.

### Mice and infection

Age- and sex-matched non-obese diabetic (NOD) mice born and bred within our animal facility were used for all experiments. Mice were housed in OptiMouse cages with corn cob bedding and up to 5 cage-mates. Mice had ad libitum access to chow and reverse osmosis chlorinated (2-3ppm)-purified water. Housing rooms were kept on a 14.5/9.5-hour light/dark cycle with temperature maintained at 22-25°C and 50-70% humidity. Sentinel mice were placed on dirty bedding and nesting material in experimental rooms and subsequently tested on a quarterly basis for presence of parasites (pinworms and fur/follicular mites, *Pneumocystis* spp. (*carinii, murina*)), bacteria (*Mycoplasma pulmonis*), and viruses (RADIL Comprehensive Panel) including Mouse Hepatitis Virus (MHV), Mouse Minute Virus (MMV), Mouse Parvovirus (NS1 – Generic Parvovirus & MPV 1-5), Theiler’s Murine Encephalitis Virus (TMEV), Epizootic Diarrhea of Infant Mice (EDIM), Sendai Virus, Pneumonia Virus of Mice (PVM), Reo3 virus (REO3), Lymphocytic Choriomeningitis Virus (LCMV), Ectromelia virus, Murine Adenovirus I/II (MAV1/MAV11), and Polyomavirus. All experiments were performed in compliance with protocols approved by the Animal Care Committee of The University of British Columbia.

Virus stocks for coxsackievirus B4 Edwards strain 2 (CVB4) were prepared as previously described (15). Normoglycemic NOD mice at 11-12 weeks old were injected intraperitoneally with either 400 plaque-forming units (pfu) CVB4 or DMEM vehicle. Non-fasting blood glucose was monitored using a OneTouch LifeScan monitor. Mice were considered diabetic with two consecutive readings above 16.2 mg/dL separated by 24 hours.

### Histology

For histological analysis, pancreata were placed in 10% neutral buffered formalin for 20-24 hours and moved to 70% ethanol before paraffin embedding. Longitudinal sections were sliced 5μm thick and stained with hematoxylin-eosin. Degree of insulitis (immune infiltration) was graded where peri-insulitis was < 25% of the islet infiltrated, partial insulitis was 25-75%, and invasive insulitis was >75%. Representative images for insulitis are supplied in **Figure S1**.

Portions of the proximal colon were fixed in methanol-Carnoy’s reagent (60% methanol, 30% chloroform, 10% glacial acetic acid) for 18-20 hours before moving tissues to 70% ethanol and sent for paraffin embedding. Cross sections were sliced at 6μm thick and stained with Alcian blue. The width of the mucus layers was measured with ImageJ software.

### Bacterial quantification

Fecal pellets were collected at specified intervals following virus infection (0, 2, 3, 7, 14, and 21 days post-infection (dpi)) and frozen immediately on dry ice before being moved to -80°C for storage. Luminal contents of the intestines were collected by flushing out the ileum and the proximal colon using sterile water. Contents were spun at 6000*g* for 15 minutes at 4°C. DNA was extracted and purified from the resulting pellets using the DNeasy PowerSoil kit (Qiagen), as per manufacturer’s instructions. Purified DNA was stored at -20°C prior to sequencing.

Bacterial DNA was quantified by quantitative polymerase-chain reaction (qPCR) using the SsoFast™ EvaGreen assay (Bio-Rad) and a CFX96 Real-Time Detection System (Bio-Rad). 16S rRNA gene sequences were targeted using bacterial-specific primers, 27F, (5′-AGAGTTTGATCCTGGCTCAG) and DW519R (5′-GNTT TACCGCGGCKGCTG). Standards for total bacterial quantification were derived from 16S rRNA gene clone libraries using a 16S rRNA gene from the bacteria SUP05 according to Zaikova et al. (65). Bacterial DNA standards, ranging from 10^2^-10^8^ copies and non-template controls were all run in duplicate. *Bifidobacteria* specific 16S rRNA genes were targeted using primers Bifido5, (5’-GATTCTGGCTCAGGATGAACGC) and Bifido3 (5’-CTGATAGGACGCGACCCCAT) (66). Standards for total quantification were made as in (66), but using a Bifidobacteria 16S rRNA gene amplified from mouse fecal material.

### Microbial community analyses

Bacterial and archaeal small subunit (SSU) rRNA gene (rDNA) fragments from the extracted genomic DNA were amplified using primers 515F and 806R (67). Sample preparation for amplicon sequencing was performed as described previously (67, 68). The amplicon library was analyzed on an Agilent Bioanalyzer using a high-sensitivity dsDNA assay to determine approximate library fragment size and to verify library integrity. Pooled library concentration was determined using the KAPA library quantification kit for Illumina. Library pools were diluted to 4 nM and denatured into single strands using fresh 0.2 N NaOH as recommended by Illumina. The final library was loaded at a concentration of 8 pM, with an additional PhiX spike-in of 5 to 20%. Sequencing was conducted on an Ilumina miseq.

### Bioinformatic analysis

Sequences were processed using the Quantitative Insights Into Microbial Ecology 2 (QIIME 2) software package (69). Denoising, chimera checking, and clustering were performed using the Divisive Amplicon Denoising Algorithm 2 (DADA2) plugin tool and denoise-paired instruction (70). For taxonomic annotation, the SILVA database (release_138) was used as the reference (71), together with the naïve-Bayes-algorithm-based trained classifier for a taxonomic assignment at 99%, using feature classifier classify-sklearn instructions (https://docs.qiime2.org/2022.2/data-resources/). All data were visualized in R, Excel and GraphPad Prism. For α- and β-diversity measures, all samples were subsampled to the lowest coverage depth and standard indices were calculated in Qiime2. ASV table was imported into the program Mothur (72) to conduct further microbial community analysis. A multiple-sample analysis of molecular variance (AMOVA) was used to test the significance of differences between microbial communities, and a statistical analysis, based on Linear discriminant analysis Effect Size (LefSe) (73), was used to test for more nuanced differences in microbial community compositions. Raw sequencing data are available under NCBI BioProject ID PRJNA855481.

### Intestinal permeability assay

Mice were fasted overnight and gavaged the next morning with 80 mg of 3000-5000 kDa FITC-dextran (Sigma-Aldrich) per 100 g bodyweight. Food was placed back in cages and blood samples were collected 4 hours later by either heart puncture or saphenous vein bleed. Blood was spun at 8000*g* for 13 minutes at room temperature (RT). Cleared serum was diluted 1:1 with sterile PBS and measured fluorometrically using a VarioSkan (Thermo Fisher Scientific) plate reader with an excitation of 485 nm and an emission wavelength of 530 nm. Samples were compared to serially diluted FITC-dextran in mouse serum to determine concentration.

### Fecal antigen ELISA

To produce sterile fecal antigen, fecal pellets were collected from naïve NOD mice, homogenized in sterile PBS, strained through a 70 μm filter, and heat killed at 70°C for 10 minutes. Fecal antigen concentration was determined using Pierce BCA Protein Assay Kit (Thermo Fisher Scientific). Immulon IV ELISA (Nunc) plates were coated with 10 μg/mL of fecal antigen in Coat Buffer (0.05M Na_2_CO_3_, 0.05M NaHCO_3_, pH 9.6) overnight at 4°C. Plates were washed with 0.05% Tween-20 in PBS and blocked with 5% fetal bovine serum (FBS) in PBS for 1 hour at 37°C. Serum samples collected from naïve NOD mice and CVB4-infected mice were diluted to either 1:50 and 1:100, added to the plates, and incubated for 2 hours at 37°C. Plates were washed and stained with HRP α-mouse IgM, IgG, IgG1, IgG2a, or IgA (Thermo Fisher Scientific) for 1 hour at 37°C. Plates were washed and TMB substrate was added. Reaction was stopped using 2N H_2_SO_4_ and read immediately at 450 nm on a VarioSkan Plate Reader (Thermo Fisher Scientific).

### Gene expression analysis

Tissue samples from pancreas, lymph nodes, ileum, and proximal colon were harvested and immediately placed in RNAlater (Qiagen). Samples were kept at 4°C overnight before transferring to -80°C for storage. Samples were thawed and RNA extracted using RNeasy RNA extraction kit (Qiagen) according to manufacturer instructions. Complimentary DNA was made using High-Capacity cDNA Reverse Transcription kit (Qiagen). qPCR reactions were carried out using iQ SYBR Green Supermix (Bio-Rad) and gene expression was measured on a CFX96 Real-Time PCR thermocycler (Bio-Rad). Primers used for qPCR reactions are listed in **Table S6**. All samples were measured in triplicate. Cycle conditions were followed by melt curve analysis to determine amplicon specificity. Gene expression was normalized to *Gapdh* and is expressed as fold-induction (calculated using the ΔΔCt method) relative to naïve mice.

### LPS assay

Serum from mice was diluted 1:75 in sterile pyrogen-free water and heat-shocked at 70°C for 15 minutes. Sample abundance of bacterial lipopolysaccharide (LPS) was measured using a Pierce Chromogenic Endotoxin Quant Kit (Thermo Fisher Scientific) according to manufacturer’s instructions. Standard curve using a known amount of endotoxin was used to determine sample concentration.

### Fecal microbiome transfers

Fecal microbiome transfer (FMT) was performed as described in Staley et al. (74). Briefly, 6-week-old NOD mice were placed on a broad-spectrum antibiotic course: “non-absorbable” antibiotics for 7 days, 2 days normal drinking water, “absorbable antibiotics” for 7 days, 2 days normal drinking water, “non-absorbable” antibiotics for 7 days, and finally 2 days on normal drinking water before receiving the FMT. “Non-absorbable” antibiotic cocktail included 1mg/mL of each ertapenum (Invanz), neomycin sulfate, and vancomycin hydrochloride. “Absorbable” antibiotic cocktail included 1 mg/mL of each ampicillin sodium salt, cefoperazone sodium salt, and clindamycin hydrochloride.

Donor samples were collected from age- and sex-matched normoglycemic NOD mice which had either been mock-infected or infected with 400 pfu CVB4 at 11-weeks-old and samples were collected at day 14 pi. Donor samples were prepared by homogenizing previously frozen fecal pellets pooled from 4-5 mice in sterile PBS at approximately 1 mg/mL in an anaerobic chamber (5% hydrogen, 95% nitrogen). Homogenized samples were passed through a 70 μm filter to remove debris and centrifuged at 6000*g* for 15 minutes at RT. Supernatant was removed and the pellet resuspended. Bacterial cells were counted using a Beckman-Coulter cell counting chamber and recipient mice gavaged with approximately 10^10^ cells. In total, we collected samples and performed FMT with 4 different naïve vs CVB4-infected donor pairs.

To check for virus presence in FMT donor samples, RNA was extracted and cDNA was made as previously described above. A semi-nested PCR was used to detect viral genes. PCR amplification was first performed using forward primer (EV1: 5′-GAGTATCAATAAGCTGCTTG-3′) and reverse primer (EV2: 5′-ATTGTCACCATAAGCAGCCA-3′) generating a 414bp fragment. This template was subjected to a secondary amplification using the EV2 reverse primer and an internal primer (EV3: 5′-TCCTCCGGCCCCTGAATGCG-3′) to produce a 155bp product. None of the donor samples indicated any positivity for virus. Pancreatic samples from FMT recipients were also checked for virus pathology histologically by looking for necrosis of acinar tissue.

### Isolation of lymphocytes for flow cytometry

Single cell suspensions from spleen, pancreatic lymph nodes (PLN), and ileum were prepared for flow cytometry analysis. Spleen and lymph nodes were mashed through 70 μm cell strainer. Spleen red blood cells were lysed with ammonium-chloride-potassium (ACK) lysis buffer for 10 minutes and washed before being resuspended in sterile FACS buffer (PBS with 2%FBS and 2mM EDTA). Free fat and Peyer’s patches were removed from small intestines. The tissue was cut open longitudinally, washed in PBS and placed in cold DMEM with 2% FBS. To remove epithelial cells, intestines were incubated in strip buffer (PBS with 1 mM EDTA, 1 mM dithiothreitol, and 5% FBS) and shaken at 180 rpm for 10 minutes at 37°C. Tissues were washed, and again incubated in strip buffer with shaking at 180 rpm for 20 minutes at 37°C. Supernatant was discarded, and tissue digested by incubation in DMEM containing 2% FBS, 0.5 mg/mL collagenase/dispase, and 0.02 mg/mL DNase I for 30 minutes at 37°C with shaking at 180 rpm. Supernatant containing lamina propria cells was passed through a 70 μm cell strainer and resuspended in FACS buffer.

### Stimulation and staining of cells for flow cytometry

Cells were stimulated with PMA (10 ng/mL) and ionomycin (500 ng/mL) in the presence of GolgiPlug (1X) and 10% FBS in MEM media for 3-4 hours at 37°C followed by viability using e506 Fixable Viability Dye (eBioscience). Cells were incubated with CD16/CD32 Fc block and extracellular markers were stained with monoclonal antibodies for 20 minutes at 4°C using the following antibodies: CD45 (30-F11), TCRβ (H57-597), CD4 (RM4-5), CD8 (53-6.7), CD19 (1D3). Cells were permeabilized using Foxp3/Transcription Factor Staining kit (eBioscience) and intracellular markers were stained with monoclonal antibodies for 45 minutes at 4°C with FoxP3 (FJK-16S) and IL-10 (JES5-16E3) (All antibodies were from Thermo Fisher Scientific). Cells were resuspended in PBS with 2% NCS and 2 mM EDTA. Samples were collected using Attune NxT Flow Cytometer (Thermo Fisher Scientific) and data analyzed with FlowJo Software (BD Biosciences).

### Statistical analysis

Statistics were performed in GraphPad Prism 9.0 software. For comparison of two normally-distributed data sets a two-tailed, unpaired T test with Welch’s correction was used for analysis. For three or more experimental groups, a One-Way ANOVA with Tukey’s multiple comparison analysis was used to determine differences. If Brown-Forsythe ANOVA test indicated an unequal variance in standard deviation of experimental groups being compared, a Welsh’s ANOVA with Dunnett’s T3 multiple comparisons test was used. A Log-rank Mantel-Cox test was used for analysis of incidence curves. Longitudinal analysis between at least two different groups was analyzed by Two-Way ANOVA with Šidák’s multiple comparisons test. Bar plots are represented as mean with standard error (SEM), and box and whisker plots indicate median with range unless indicated otherwise in figure legends. Significance is indicated with asterisks, where *p<0.05, **p<0.01, ***p<0.001, ****p<0.0001, and n.s. = not significant.

## Data availability statement

The datasets presented in this study can be found in online repositories. The names of the repository/repositories and accession number(s) can be found in the article/**Supplementary Material**.

## Ethics statement

The animal study was reviewed and approved by Animal Care Committee of The University of British Columbia.

## Author contributions

Conceptualization: ZM, RS, SC, MH, LO. Methodology: ZM, RS. Investigation: ZM, RS. Visualization: ZM, RS. Supervision: SC, MH, LO. Writing, original draft: ZM, LO. Writing, review & editing: ZM, RS, SC, MH, LO. All authors contributed to the article and approved the submitted version.
