# Supplementary material for: Virus induced dysbiosis promotes type 1 diabetes onset
Source: Front Immunol. 2023 Jan 19;14:1096323. doi: 10.3389/fimmu.2023.1096323 (PMC9892191; doi:10.3389/fimmu.2023.1096323)
Supplement: Supplementary file 1 [file DataSheet_1.pdf]

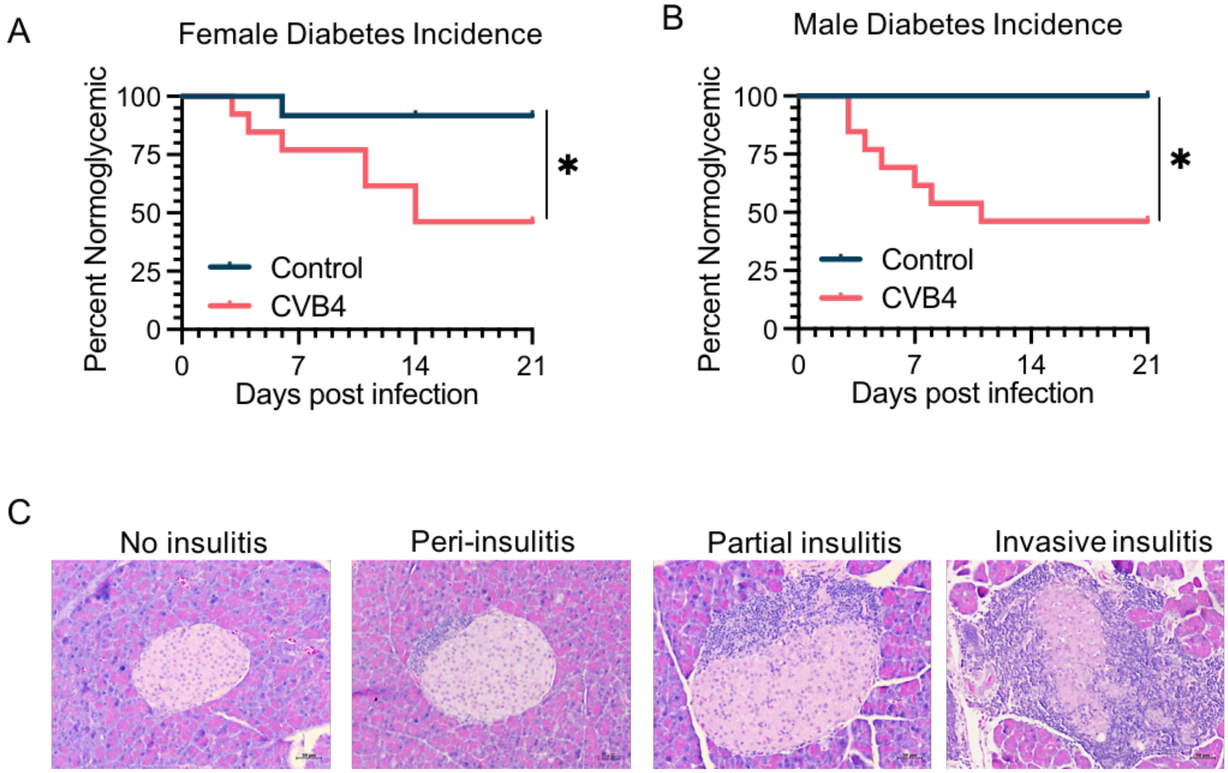

Figure S1. CVB4 induction of T1D is sex-independent. Diabetes incidence in (A) female (n = 12-13 mice per group) and (B) male (control n = 8, CVB4 n = 13) NOD mice following CVB4 infection. Data are combined from 3 independent experiments and analyzed using a log-rank (Mantel-Cox) test. (C) Representative images for insulitis scoring. * $P \leq 0.05$ was considered statistically significant.

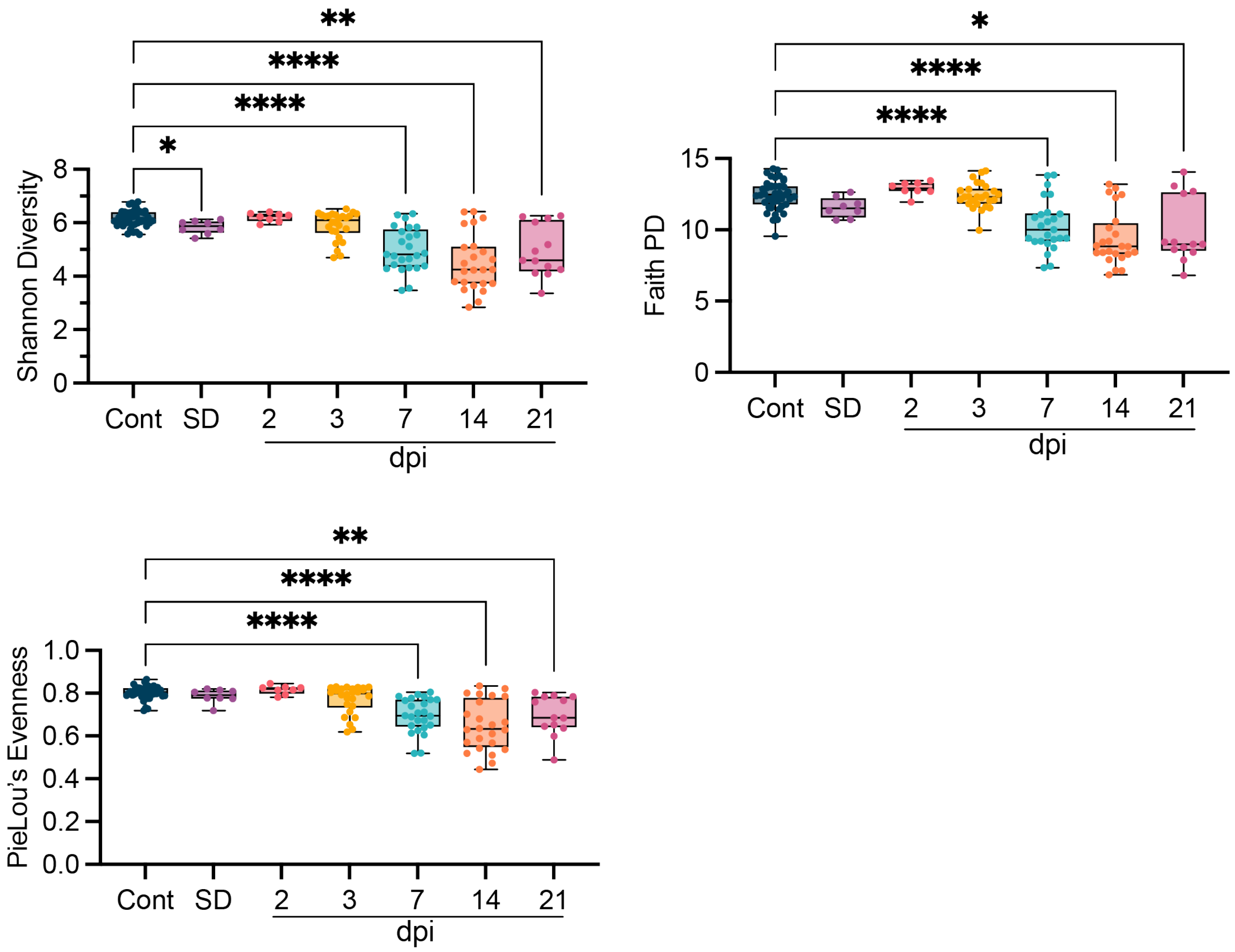

Figure S2. CVB4 Infection reduces α-diversity of intestinal microbiome. Additional α-diversity measures over the course of CVB4 infection. P values were calculated using Welch's ANOVA with Dunnet's T3 multiple comparisons test. * $P \leq 0.05$ was considered statistically significant; ** $P \leq 0.01$, **** $P \leq 0.0001$.

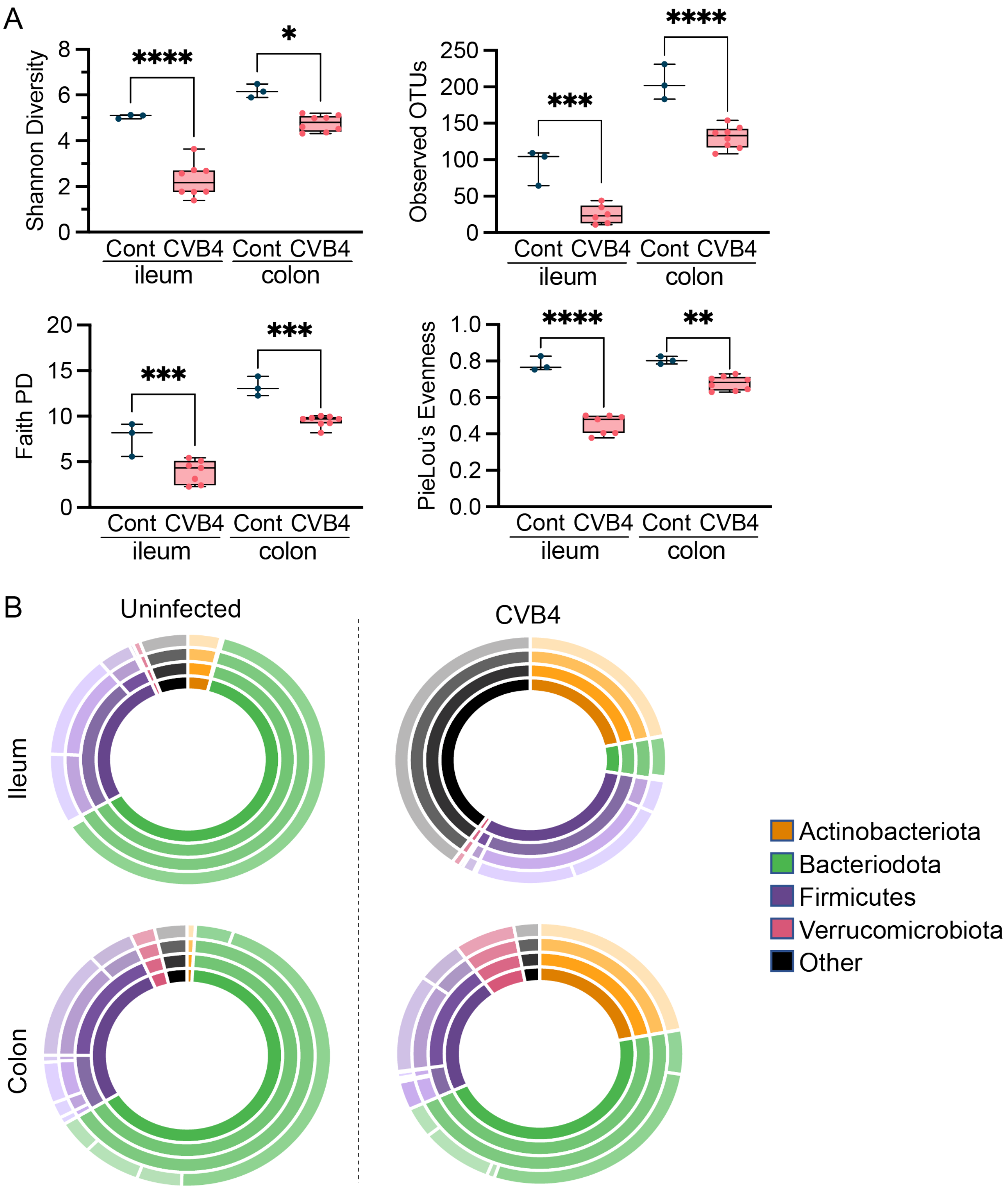

Figure S3. Changes in the fecal microbial composition are reflected in the luminal contents of the small and large intestine. (A) α-diversity measures of luminal contents taken from the ileum and colon at day 14 pi. (B) Microbial composition of luminal contents. Inner rings indicate composition on a phylum level and each subsequent ring represents a lower taxonomic level (class, order, family). (Uninfected n = 3, CVB4 n = 8). Data were analyzed using Welch's *t* test (two-tailed). * $P \leq 0.05$ was considered statistically significant; ** $P \leq 0.01$, *** $P \leq 0.001$; **** $P \leq 0.0001$.

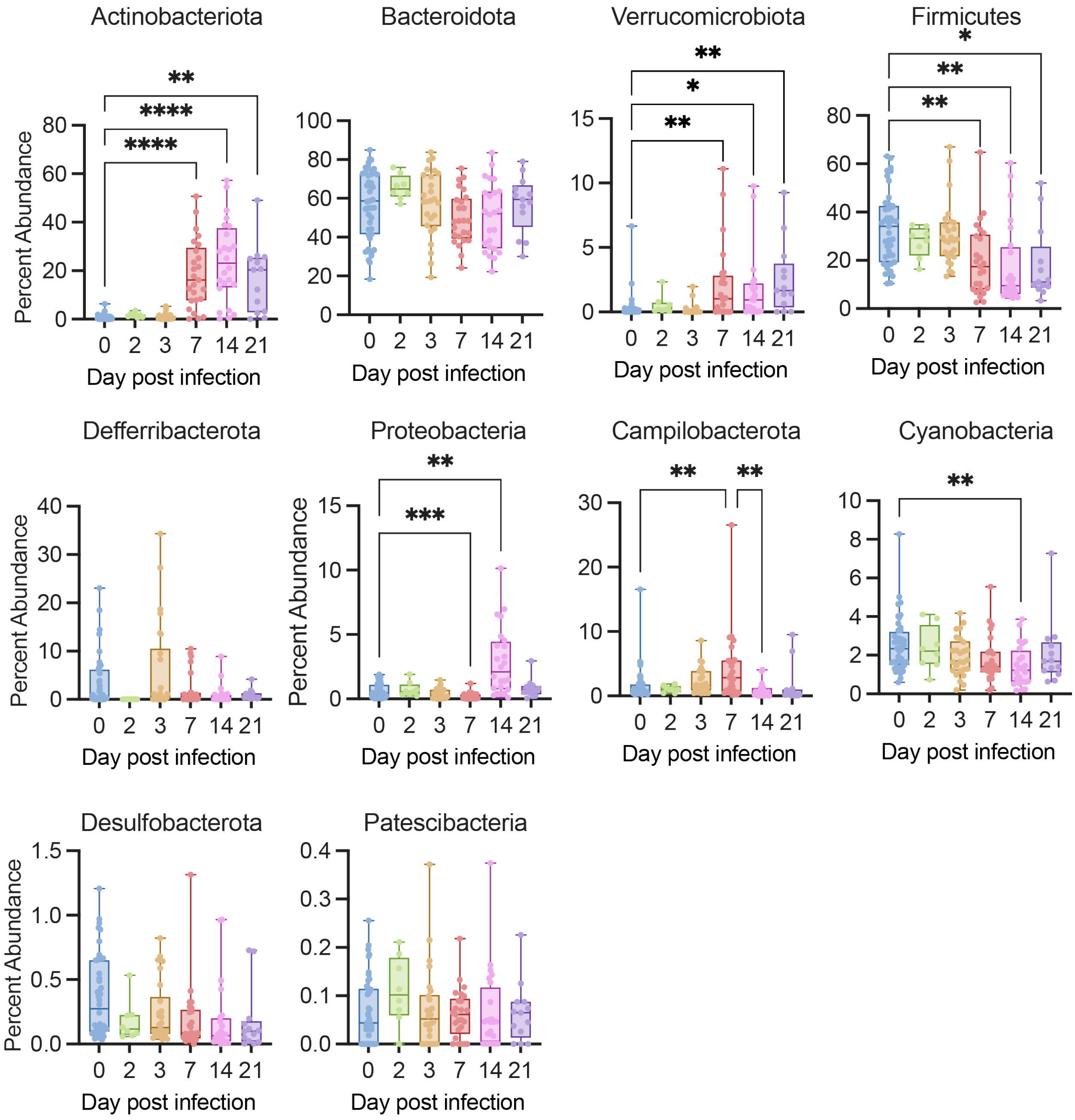

Figure S4. Relative abundance of microbial phyla in fecal pellets throughout CVB4 infection. Results are representative of three independent experiments. A total of n = 26 mice were sampled longitudinally following infection with CVB4. Some mice were harvested at day 14 pi for analysis of luminal contents of the intestine all others were harvest at day 21 pi. P values were calculated using Welch's ANOVA with Dunnet's T3 multiple comparisons test. * P ≤ 0.05 was considered statistically significant; ** P≤ 0.01, *** P≤ 0.001; **** P≤ 0.0001.

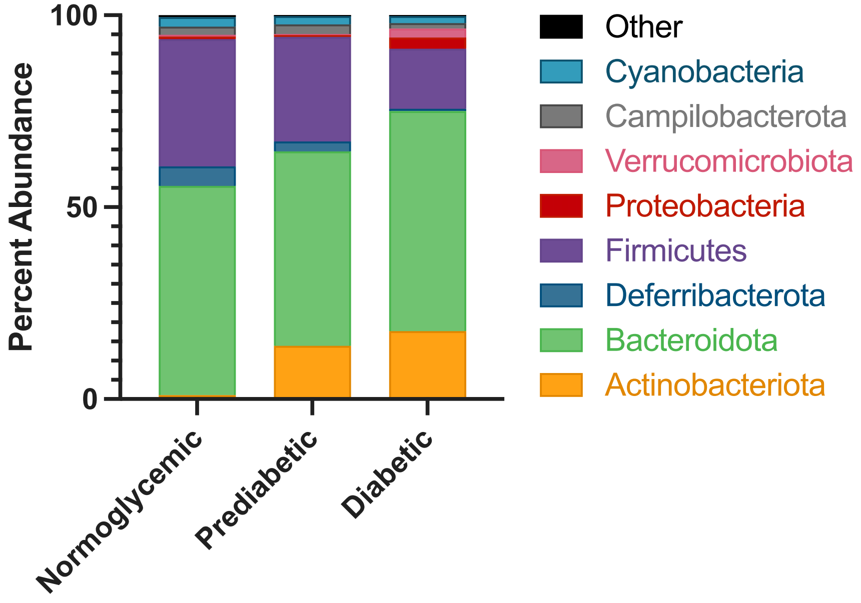

Figure S5. The microbiome shifts prior to diabetes onset. In the subset of mice which are infected and go on to become diabetic, 16S community profiles of fecal pellets collected from CVB4-infected mice were grouped based on blood sugar levels rather than day of infection: Normoglycemic (<16.2 mg/dL), Diabetic (>16.2 mg/dL). The Prediabetic group consists of fecal samples collected from mice on the day of the last "Normoglycemic" reading. The characteristic dysbiotic community composition is apparent prior to hyperglycemia. n = 14 mice.

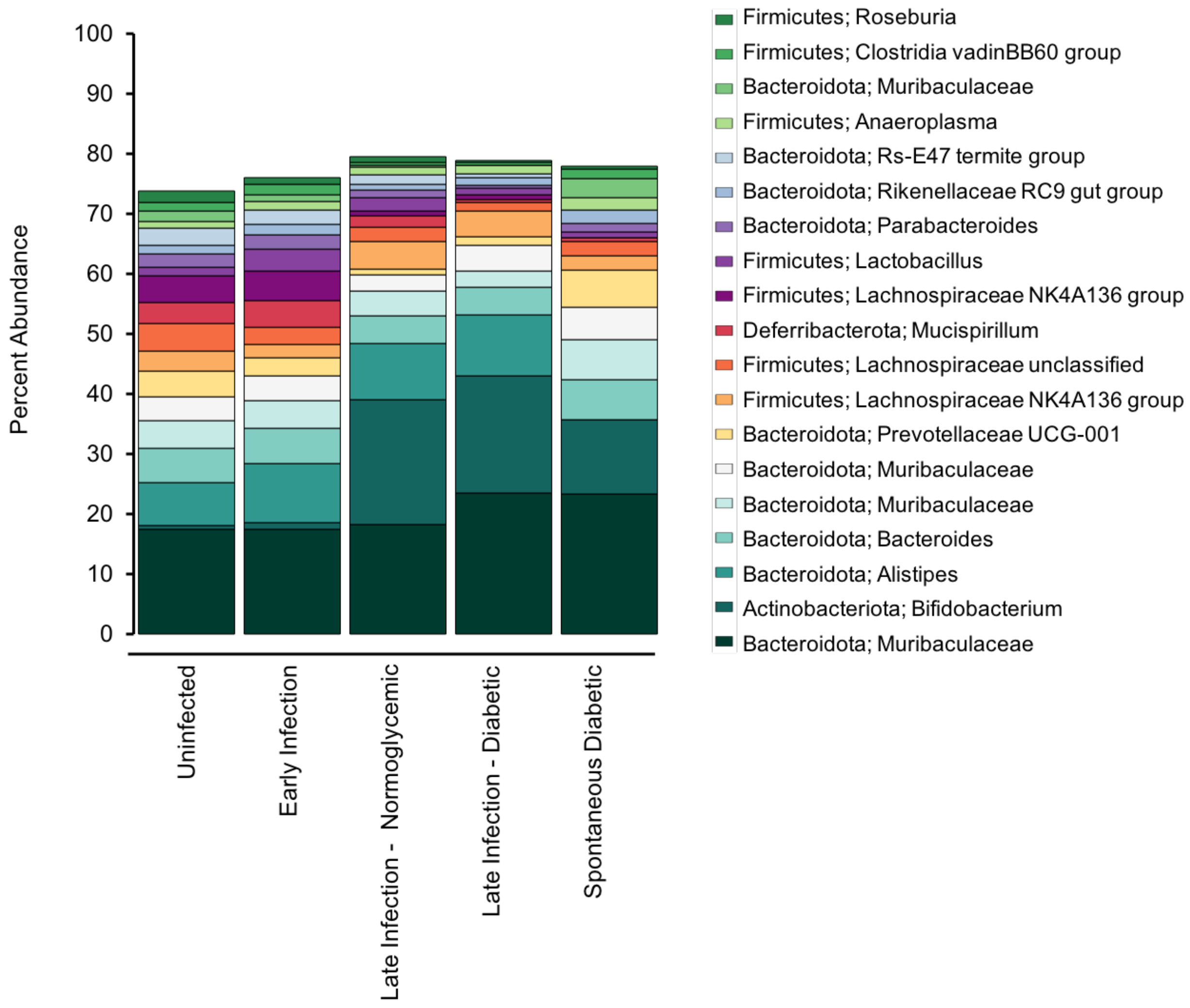

Figure S6. Bacterial genera in fecal microbial communities. Uninfected (normoglycemic, days 0-21 pi, n = 72); Early infection, days 0-3 pi (n = 34); Late infection - Normoglycemic, days 7-21 pi with blood sugar <16.2 mg/dL (n = 38); Late infection – Diabetic, days 7-21 pi with blood sugar >16.2 mg/dL (n = 24); Spontaneous diabetic, no infection with blood sugar >16.2 mg/dL (n = 8).

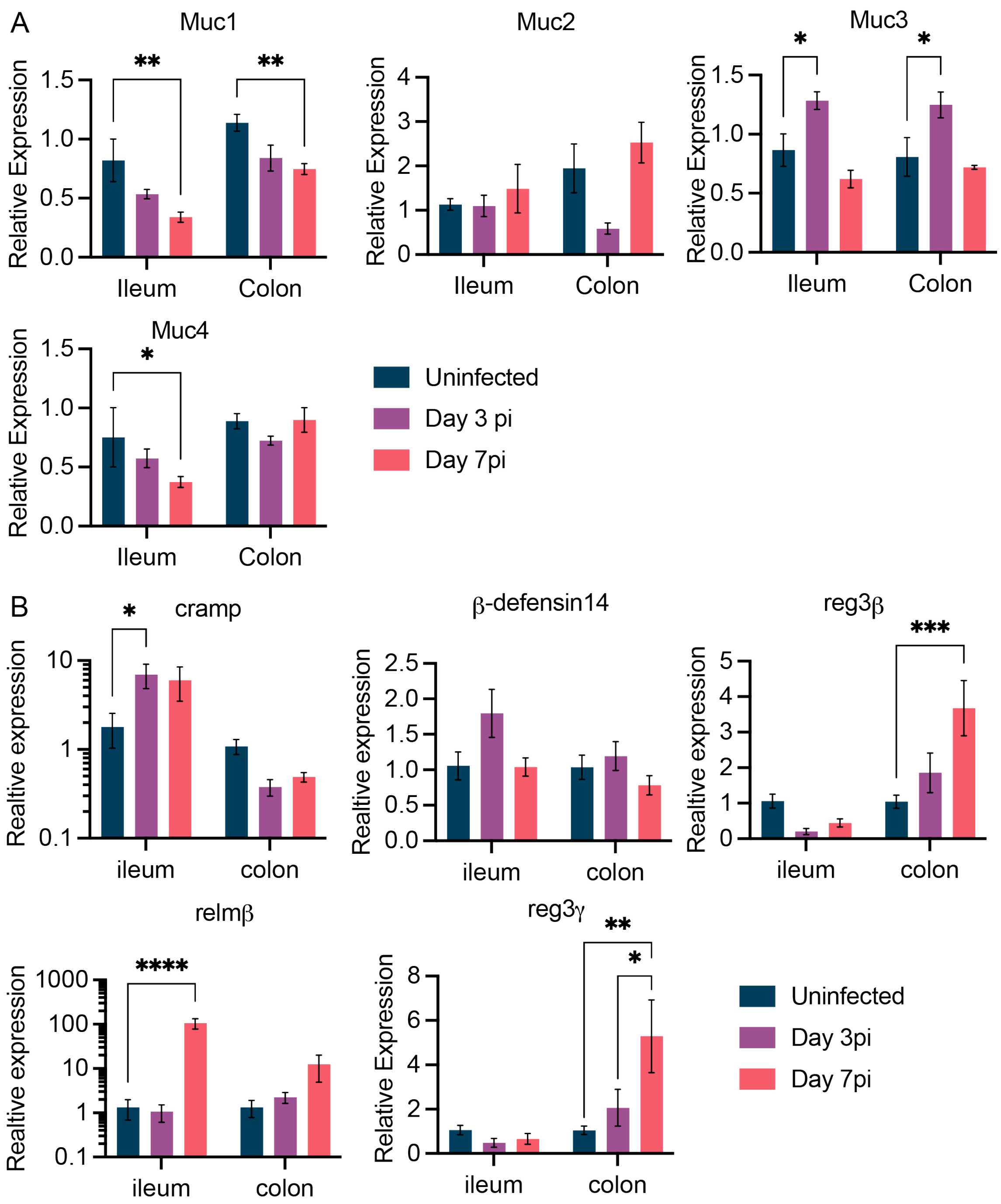

Figure S7. CVB4 infection alters intestinal expression of mucin and antimicrobial peptide genes. Expression of genes encoding (A) mucins and (B) antimicrobial peptides in the ileum and proximal colon of naïve and CVB4-infected mice as determined by RT-qPCR. All qPCR reactions are normalized to expression of GAPDH and represented as the relative expression to uninfected control mice (n = 3-4 mice per group). P values were calculated using Welch's ANOVA with Dunnet's T3 multiple comparisons test. Results are expressed as the mean ± SEM. *, P ≤ 0.05 was considered statistically significant; **, P≤ 0.01; ***, P≤ 0.001; ****, P≤ 0.0001

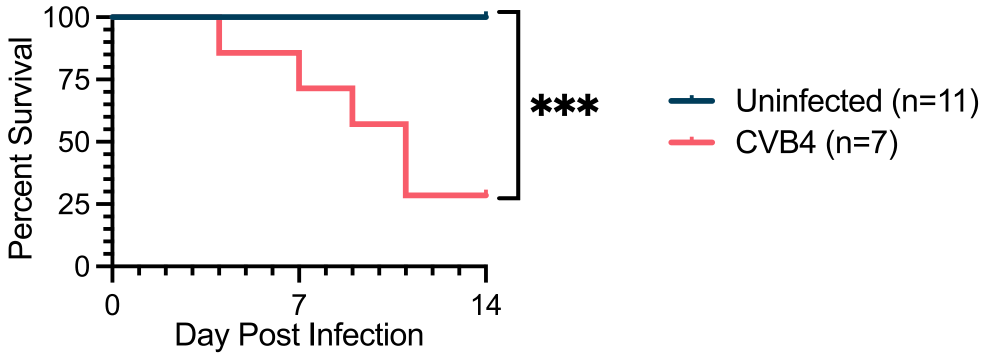

Figure S8. The microbiome is necessary for survival following CVB4 infection. NOD mice (6 weeks) were depleted of their endogenous microbiota using antibiotics as described in Figure 5A before being infected with 400pfu CVB4 intra-peritoneally at 11 weeks old. Survival was monitored for 2 weeks post-infection. Data are from a single experiment and analyzed using a log-rank (Mantel-Cox) test. ***, $P \leq 0.001$

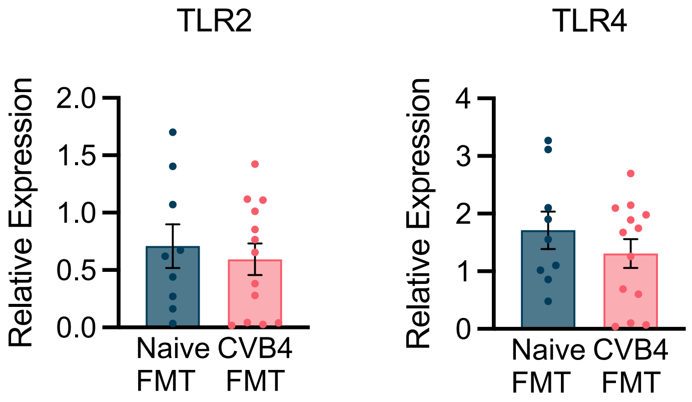

Figure S9. CVB4 infection does not affect expression of bacterial innate receptors. TLR expression in the proximal colon of FMT recipient mice at day 35 post-FMT. The results are representative of three independent experiments and expressed as the mean ± SEM. Data were analyzed using Welch's t test (two-tailed).

Table S1. Analysis of Molecular Variance (AMOVA) display differences in sampling timepoints following CVB4 infection. Bacteria compositional differences at various timepoints were compared in (A) uninfected and (B) CVB4-infected mice. The p values indicating dissimilarity in community structure between individual timepoints are represented. *** = $p < 0.001$. DPI = days post infection

A

		Uninfected DPI				
		0	3	7	14	21
Uninfected DPI	0		0.11	0.15	0.12	0.11
	3			0.94	0.91	0.17
	7				0.99	0.21
	14					0.13
	21					

B

		CVB4 DPI				
		0	3	7	14	21
CVB4 DPI	0		0.58	***	***	***
	3			***	***	***
	7				0.02	0.03
	14					0.39
	21					

Table S2. Microbial community composition differs among experimental groups. Analysis of Molecular Variance (AMOVA) analysis comparing microbial population differentiation based on infection, time point, and glycemia. Control = uninfected, days 0-21 pi, normoglycemic (n=72); Early CVB4 Infection = Days 0-3 pi (n=34); Late CVB4 Infection = Days 7-21 pi (n=62 total, of which n=34 normoglycemic and n=24 diabetic); SD = naïve, spontaneously diabetic (n=8).

Control - SD			
	Among	Within	Total
SS	0.39448	7.35595	7.75043
df	1	78	79
MS	0.39448	0.094307	
Fs:	4.18294		
p-value:	**<0.001***		

Early CVB4 Infection - SD			
	Among	Within	Total
SS	0.43974	3.70058	4.14032
df	1	40	41
MS	0.43974	0.0925146	
Fs:	4.75319		
p-value:	**<0.001***		

Control - Early CVB4 Infection			
	Among	Within	Total
SS	0.180538	10.2895	10.47
df	1	104	105
MS	0.180538	0.0989371	
Fs:	1.82478		
p-value:	**0.013**		

Late CVB4 Infection - SD			
	Among	Within	Total
SS	0.77889	11.8667	12.6456
df	1	68	69
MS	0.77889	0.17451	
Fs:	4.46329		
p-value:	<0.001*		

Control - Late CVB4 Infection			
	Among	Within	Total
SS	2.7012	18.4556	21.1568
df	1	132	133
MS	2.7012	0.139815	
Fs:	19.3198		
p-value:	**<0.001***		

Diabetic - Normoglycemic			
	Among	Within	Total
SS	1.3269	24.5252	25.8521
df	1	174	175
MS	1.3269	0.14095	
Fs:	9.41399		
p-value:	**<0.001***		

Early CVB4 Infection - Late CVB4 Infection			
	Among	Within	Total
SS	1.71708	14.8002	16.5173
df	1	94	95
MS	1.71708	0.157449	
Fs:	10.9056		
p-value:	**<0.001***		

"Late Infection - Diabetic" vs "Late Infection - Normoglycemic"			
	Among	Within	Total
SS	0.47	11.02	11.49
df	1	60	61
MS	0.47	0.18	
Fs:	2.55		
p-value:	0.004		

Table S3. Indicator species from LEfSe analysis on microbial composition of “Uninfected” control mice.

ASV ID	Taxonomy	LogMax Mean	LDA	pValue
f80166d86a7f15b69b6ac97505299c3a	Bacteroidota; Bacteroidia; Bacteroidales; Muribaculaceae; Muribaculaceae; uncultured_bacterium	4.54	3.97	4.2E-03
0ebb2cf4a2017aeaefadf9ae5062eb62	Bacteroidota; Bacteroidia; Bacteroidales; Muribaculaceae; Muribaculaceae;	4.42	3.55	8.0E-03
08a568a23077f42668f7b4b5e0ce437e	Bacteroidota; Bacteroidia; Bacteroidales; Muribaculaceae; Muribaculaceae; uncultured_bacterium	4.19	3.80	1.9E-03
b44aff2995f396f00ad579d8e6684335	Bacteroidota; Bacteroidia; Bacteroidales; Muribaculaceae; Muribaculaceae; uncultured_bacterium	4.15	3.66	1.1E-11
e4597f1dce864c29882fa64f616d1a6c	Bacteroidota; Bacteroidia; Bacteroidales; Muribaculaceae; Muribaculum; uncultured_bacterium	4.14	3.76	0.0E+00
2eb2fe0471e37d54b5e1c47660220ad6	Bacteroidota; Bacteroidia; Bacteroidales; Muribaculaceae; Muribaculaceae; uncultured_bacterium	3.99	3.50	2.2E-12
cb8b8a6ee6bcfa115f8b24a45fa1c12a	Bacteroidota; Bacteroidia; Bacteroidales; Muribaculaceae; Muribaculaceae; uncultured_Bacteroidales	3.87	3.41	3.2E-13
9522a330db4a497684cae267c651eb30	Bacteroidota; Bacteroidia; Bacteroidales; Muribaculaceae; Muribaculaceae;	3.86	3.07	2.6E-03
b358d76cf51dad99d526470074e5f31c	Firmicutes; Clostridia; Lachnospirales; Lachnospiraceae; Lachnospiraceae_NK4A136_group;	3.91	3.41	1.3E-05
270a745647e1f4f2daefddcae78b43c7	Firmicutes; Clostridia; Lachnospirales; Lachnospiraceae; uncultured;	3.81	3.25	4.5E-07
cdb79eecfa38ac58e99bfdef45fabfce	Firmicutes; Clostridia; Lachnospirales; Lachnospiraceae; Lachnospiraceae_NK4A136_group;	3.85	3.34	1.4E-04
37a0a427f45ea3b8299b4a41c10d02e6	Firmicutes; Clostridia; Lachnospirales; Lachnospiraceae; ;	3.82	3.28	1.6E-07
7421a0056850e30172e2c541c9215c11	Proteobacteria; Gammaproteobacteria; Burkholderiales; Sutterellaceae; Parasutterella; uncultured_bacterium	3.74	3.37	6.0E-13
6c921e3ae0af506f54295d074ca071bc	Firmicutes; Clostridia; Lachnospirales; Lachnospiraceae; A2;	3.65	3.13	4.8E-10
2f38713af6dbed30ebf9709d2d9aaa5e	Firmicutes; Clostridia; Lachnospirales; Lachnospiraceae; Lachnospiraceae_NK4A136_group;	3.83	3.43	1.1E-16
25f537c4856de1617e682de90caba215	Firmicutes; Clostridia; Lachnospirales; Lachnospiraceae; Lachnospiraceae_NK4A136_group;	3.75	3.32	3.4E-07
13506518b825bfbd65794347af5958c6	Firmicutes; Clostridia; Lachnospirales; Lachnospiraceae; Roseburia; uncultured_bacterium	3.76	3.41	3.6E-10
b1652dc664d4e8ce2d123d26687d1204	Firmicutes; Clostridia; Lachnospirales; Lachnospiraceae; [Eubacterium]_xylanophilum_group; uncultured_bacterium	3.68	3.26	8.8E-13
acf76b1f22c9536ca982df6f9b0219da	Firmicutes; Clostridia; Lachnospirales; Lachnospiraceae; Roseburia; uncultured_bacterium	3.63	3.20	2.7E-03
4a18a72af06424cd6600b6f548ea8f05	Firmicutes; Clostridia; Oscillospirales; Ruminococcaceae; Ruminococcaceae; uncultured_bacterium	3.59	3.15	1.9E-08
e04cb8c96d35fee0e181a15fc4511d0c	Firmicutes; Clostridia; Lachnospirales; Lachnospiraceae; ;	3.59	3.20	2.2E-14
35b8c10c192360a7cdad628a185e99e5	Firmicutes; Clostridia; Lachnospirales; Lachnospiraceae; Lachnospiraceae_NK4A136_group; uncultured_bacterium	3.53	3.13	4.7E-10
6b4bd54750d579153464bf73cdb8dfd1	Firmicutes; Clostridia; Lachnospirales; Lachnospiraceae; Lachnospiraceae_NK4A136_group; uncultured_bacterium	3.55	3.09	1.0E-04
d3d5f57b1c8aafeca61afdb1627d2990	Firmicutes; Clostridia; Lachnospirales; Lachnospiraceae; Roseburia;	3.47	3.12	4.3E-09
4e0a65b0a2fab7bf0f307d360b679d87	Firmicutes; Clostridia; Lachnospirales; Lachnospiraceae; ;	3.46	3.07	3.9E-13
84c7caa8ea59cf2cb4450742af1e088b	Bacteroidota; Bacteroidia; Bacteroidales; Muribaculaceae; Muribaculaceae; uncultured_bacterium	3.38	2.87	1.9E-08
6a8118b17d1d40cf877db24449ceb616	Firmicutes; Clostridia; Oscillospirales; Oscillospiraceae; Oscillibacter;	3.41	2.95	1.1E-08
f3e9d78daeea42d345080748e31ae3dd	Firmicutes; Clostridia; Oscillospirales; Oscillospiraceae; Colidextribacter;	3.35	2.93	5.0E-10
92cacd5cf65022777afd65aeeb844c27	Firmicutes; Clostridia; Lachnospirales; Lachnospiraceae; ;	3.52	3.18	0.0E+00
b9ed1a2c86edb972db7742981e6901da	Firmicutes; Clostridia; Oscillospirales; Ruminococcaceae; uncultured; [Clostridium]_leptum	3.31	2.84	8.8E-06
fded9ff7f54a838e9511e58e26c46064	Firmicutes; Clostridia; Lachnospirales; Lachnospiraceae; Roseburia; uncultured_bacterium	3.42	2.94	5.3E-07
2cf0267a31dc6f1b8ee65c913b8f6e4d	Firmicutes; Clostridia; Lachnospirales; Lachnospiraceae; uncultured; uncultured_bacterium	3.19	2.68	7.3E-04
0c10bc475287f99f197cfc1c2b50947f	Bacteroidota; Bacteroidia; Bacteroidales; Rikenellaceae; Rikenella; uncultured_bacterium	3.32	2.86	6.1E-07
d6eda88bd8370a52076b0dafde12249a	Firmicutes; Clostridia; Lachnospirales; Lachnospiraceae; Lachnospiraceae_FCS020_group; mouse_gut	3.36	2.86	6.7E-08
6337b308d49ea78bc68f9100ca6c62b4	Firmicutes; Clostridia; Clostridia_vadinBB60_group; Clostridia_vadinBB60_group; Clostridia_vadinBB60_group; uncultured_bacterium	3.25	2.91	2.4E-02
ee766857ab5fc018ac26632146bc90e1	Firmicutes; Clostridia; Oscillospirales; Oscillospiraceae; Colidextribacter; uncultured_bacterium	3.29	2.81	1.5E-10

c803726dac1aad6846d209e915cea275	Firmicutes; Clostridia; Oscillospirales; Oscillospiraceae; Oscillibacter; uncultured_bacterium	3.19	2.77	4.6E-11
1dacfff5549a7d2494f6a935ea1fb7e8	Desulfobacterota; Desulfovibrionia; Desulfovibrionales; Desulfovibrionaceae; Desulfovibrio; uncultured_bacterium	3.14	2.79	1.3E-05
d925f54f2129619db22eb998d6021440	Firmicutes; Clostridia; Oscillospirales; Ruminococcaceae; Anaerotruncus; uncultured_bacterium	3.35	2.92	3.0E-09
4e1bda045e6ba843353281138c649093	Firmicutes; Clostridia; Lachnospirales; Lachnospiraceae; Lachnospiraceae_NK4A136_group; uncultured_bacterium	3.10	2.64	8.9E-09
2197e3c697e9c1427ae1c3277d3c71f9	Firmicutes; Clostridia; Lachnospirales; Lachnospiraceae; ASF356; uncultured_bacterium	3.32	2.89	2.6E-10
0f2a5acc53e55f2c9f0c7409421dc5a0	Firmicutes; Clostridia; Oscillospirales; Ruminococcaceae; Anaerotruncus; uncultured_bacterium	3.18	2.83	1.5E-07
e046e29a358f4e382dccc33ca22f61ad	Firmicutes; Clostridia; Lachnospirales; Lachnospiraceae; Lachnospiraceae_NK4A136_group;	3.08	2.35	5.6E-04
ac28c199405fa8466bdedf16d456edbc	Firmicutes; Clostridia; Lachnospirales; Lachnospiraceae; A2; uncultured_bacterium	3.23	2.86	1.2E-04
8aeb82845cf39311d0c9a6a82b39a131	Firmicutes; Clostridia; Oscillospirales; Ruminococcaceae; uncultured; [Clostridium]_leptum	3.18	2.77	1.2E-05
a6bed81ae136bbcaf60c91d83befaa68	Firmicutes; Clostridia; Oscillospirales; Ruminococcaceae; Incertae_Sedis; uncultured_bacterium	3.18	2.73	2.8E-12
1c281deaf71c6d702d1ddadfa953eac6	Firmicutes; Clostridia; Lachnospirales; Lachnospiraceae; uncultured; unidentified	3.09	2.63	6.4E-07
501a3e687c65822914e1a727e5f43331	Firmicutes; Clostridia; Oscillospirales; Ruminococcaceae; uncultured; uncultured_bacterium	3.16	2.78	5.3E-10
958d78a02bef69a795806f97adb117ea	Firmicutes; Clostridia; Oscillospirales; Oscillospiraceae; Colidextribacter;	3.05	2.65	4.0E-08
d92d53b67f4b4a2516ca359494db4c24	Firmicutes; Clostridia; Oscillospirales; Oscillospiraceae; Oscillibacter;	3.13	2.74	2.3E-10
85db0d60e8985f5dd02b1b3c10e2c371	Firmicutes; Clostridia; Lachnospirales; Lachnospiraceae; ASF356; uncultured_bacterium	3.16	2.72	5.7E-09
65ec17bde16247dae43cc95be7729dca	Firmicutes; Clostridia; Lachnospirales; Lachnospiraceae; Lachnoclostridium;	3.05	2.74	3.2E-07
6e5be229dd49e819c917d2ae8b72610b	Firmicutes; Clostridia; Oscillospirales; Ruminococcaceae; Incertae_Sedis; uncultured_bacterium	3.11	2.72	9.8E-13
81f59f6a76c8682de1719c97b5b24173	Firmicutes; Clostridia; Oscillospirales; Butyricicoccaceae; Butyricicoccus; uncultured_bacterium	3.02	2.66	5.4E-12
df1e3080300aed496c300246714bd838	Firmicutes; Clostridia; Lachnospirales; Lachnospiraceae; Lachnospiraceae_UCG-001;	3.26	2.97	3.9E-12
91a093473cad95da81d9ebb47d5ef0c5	Firmicutes; Clostridia; Lachnospirales; Lachnospiraceae; ;	2.93	2.48	1.3E-05
273fa0191072af3d33e32271b35c8f18	Firmicutes; Clostridia; Oscillospirales; Oscillospiraceae; ;	2.99	2.51	2.4E-05
ea9c1edfdf60a0a532f516b20689e41b	Bacteroidota; Bacteroidia; Bacteroidales; Muribaculaceae; Muribaculaceae; uncultured_bacterium	2.93	2.73	1.7E-15
af140e3881d1c74ce8b17a5c8979dfa5	Firmicutes; Clostridia; Lachnospirales; Lachnospiraceae; uncultured; uncultured_bacterium	3.03	2.58	5.4E-03
46f09d7aa9bcac4a3e3e8574925eb57a	Desulfobacterota; Desulfovibrionia; Desulfovibrionales; Desulfovibrionaceae; uncultured; uncultured_Desulfovibrionales	3.00	2.66	1.1E-03
5a84cbca09090824be95118781802f82	Firmicutes; Clostridia; Oscillospirales; Oscillospiraceae; ;	2.99	2.69	4.6E-11
b2097d12843899152a99e4faeb92e3e6	Firmicutes; Clostridia; Oscillospirales; Ruminococcaceae; uncultured; uncultured_bacterium	3.02	2.66	1.3E-07
9a28e6b1aaae69477039957bc8a1222f	Firmicutes; Clostridia; Lachnospirales; Lachnospiraceae; ;	2.77	2.45	1.2E-08
741517c5e1227b3435b9fdbcfe8788e4	Firmicutes; Clostridia; Oscillospirales; Oscillospiraceae; uncultured; unidentified	3.01	2.65	1.7E-09
00cd2f686031247590474878075 89f27	Firmicutes; Clostridia; Lachnospirales; Lachnospiraceae; ;	2.93	2.59	3.8E-09
d3d0b6aba6f97f1ba77a412531587a84	Bacteroidota; Bacteroidia; Bacteroidales; Muribaculaceae; Muribaculaceae; uncultured_bacterium	2.90	2.68	7.7E-07
ce78414357068359ddae6a47b6592a08	Firmicutes; Clostridia; Oscillospirales; Oscillospiraceae; uncultured; uncultured_bacterium	2.85	2.53	1.9E-04
1e66289327edd690ec0ad509d4257101	Firmicutes; Clostridia; Oscillospirales; Oscillospiraceae; Colidextribacter;	2.87	2.59	2.5E-10
c75ae6008025d80134bd14e9712f9d5c	Firmicutes; Clostridia; Lachnospirales; Lachnospiraceae; GCA-900066575; uncultured_bacterium	2.80	2.44	1.2E-06
7a8d0125158d1dded935e4652afc68d7	Firmicutes; Clostridia; Oscillospirales; Oscillospiraceae; uncultured; uncultured_bacterium	2.83	2.49	1.5E-05
924668f542df9de35c2226e4a6cf09bd	Firmicutes; Clostridia; Lachnospirales; Lachnospiraceae; Lachnospiraceae_NK4A136_group;	2.65	2.27	3.9E-02
12470c486f558c083c46b45408cae9c6	Firmicutes; Clostridia; Oscillospirales; Butyricicoccaceae; UCG-009; uncultured_bacterium	2.85	2.59	6.2E-12
022cacfa071489a242227dc6aa9caeed	Firmicutes; Clostridia; Oscillospirales; Oscillospiraceae; uncultured; uncultured_Clostridiales	2.74	2.18	6.4E-03
d93a3ef54cd04d16c30a94e15287caf4	Firmicutes; Clostridia; Lachnospirales; Lachnospiraceae; Lachnospiraceae_UCG-001;	2.86	2.60	4.9E-08
fcc84f75257e928f79853e39bb2c6d23	Firmicutes; Clostridia; Lachnospirales; Lachnospiraceae; Lachnospiraceae_FCS020_group;	2.46	2.39	1.6E-02
f76f9398a707c47754f8308d099abd13	Firmicutes; Clostridia; Lachnospirales; Lachnospiraceae; Lachnospiraceae_UCG-001;	2.87	2.61	1.1E-07

4066e07db1bae989fd02d771401e3b09	Firmicutes; Clostridia; Oscillospirales; Oscillospiraceae; UCG-005; uncultured_Clostridiales	2.69	2.51	1.6E-04
4ac5de6617d5b4d5201a8d2d98c785e7	Firmicutes; Clostridia; Oscillospirales; Ruminococcaceae; uncultured; uncultured_bacterium	2.56	2.45	2.4E-09
f3f57fb4feeef35aada1a409a3352520	Firmicutes; Clostridia; Lachnospirales; Lachnospiraceae; Lachnoclostridium;	2.62	2.39	2.5E-05
8212472385df0dc1b6527a764d613b7d	Firmicutes; Clostridia; Lachnospirales; Lachnospiraceae; Acetatifactor; uncultured_bacterium	2.52	2.31	7.2E-05
84977ba94188c5d008f9861e3e14d0a5	Firmicutes; Clostridia; Lachnospirales; Lachnospiraceae; ;	2.54	2.51	1.7E-07
851bd80d021dcf4e9f3f11e95310927c	Firmicutes; Clostridia; Oscillospirales; Oscillospiraceae; uncultured; uncultured_bacterium	2.49	2.16	2.0E-02
7aed90fb085e16c3c98bfa68130096d0	Firmicutes; Clostridia; Oscillospirales; Oscillospiraceae; NK4A214_group;	2.36	2.35	2.8E-02
9ecb011b73b151660fc0f8650ef1da00	Firmicutes; Clostridia; Lachnospirales; Lachnospiraceae; GCA-900066575; uncultured_bacterium	2.33	2.33	1.4E-03
5eaeab6fa2a8efe96eac7a0712c65080	Firmicutes; Clostridia; Lachnospirales; Lachnospiraceae; Blautia; Lachnospiraceae_bacterium	2.29	2.15	3.2E-02
b664aba9c5e0e5cc58a311f8ff3d8b0c	Firmicutes; Clostridia; Peptococcales; Peptococcaceae; Peptococcus; uncultured_bacterium	2.26	2.25	1.2E-05
c518773de1fa8f58aebfc3c18e017d10	Firmicutes; Clostridia; Oscillospirales; Oscillospiraceae; ;	2.02	2.61	1.2E-04

Table S4. Indicator species from LEfSe analysis on microbial composition of "Early Infection" (Day 0-3 pi) mice.

ASV ID	Taxonomy	LogMax Mean	LDA	pValue
9a13cbb8e1b12468bf22d6a05aafb0c0	Bacteroidota; Bacteroidia; Bacteroidales; Rikenellaceae; Alistipes; uncultured_bacterium	4.90	4.61	1.9E-05
5292f29cab69c370997bcb039ef68d64	Bacteroidota; Bacteroidia; Bacteroidales; Bacteroidaceae; Bacteroides;	4.53	3.92	5.1E-11
476a27c3796dfc1790e181f789663a33	Firmicutes; Clostridia; Lachnospirales; Lachnospiraceae; Lachnospiraceae_NK4A136_group; uncultured_Clostridiales	4.68	4.37	2.0E-09
9cd64c55108d1c4e92a30893188038cb	Bacteroidota; Bacteroidia; Bacteroidales; Rs-E47_termite_group; Rs-E47_termite_group; uncultured_bacterium	4.38	4.09	1.1E-10
634e293920d238dfa24961d096247760	Bacteroidota; Bacteroidia; Bacteroidales; Tannerellaceae; Parabacteroides;	4.37	3.81	6.8E-15
95fdd816723ca482a5caba10bea171c8	Firmicutes; Bacilli; Lactobacillales; Lactobacillaceae; Lactobacillus;	4.16	3.71	1.8E-02
df0e3d38eec730326754d8c17a8b8efe	Firmicutes; Bacilli; Erysipelotrichales; Erysipelotrichaceae; Turicibacter;	4.01	3.57	2.3E-03
0df6c802966e8670279671824da4f10a	Firmicutes; Bacilli; Lactobacillales; Lactobacillaceae; Lactobacillus;	3.86	3.41	4.6E-04
7da4c046b2e9582e3f885b029541976c	Firmicutes; Clostridia; Clostridia_vadinBB60_group; Clostridia_vadinBB60_group; Clostridia_vadinBB60_group; unidentified	3.87	3.39	2.4E-07
ad235b65ccb597f05a96b06c0ad90eee	Bacteroidota; Bacteroidia; Bacteroidales; Rikenellaceae; Alistipes; uncultured_bacterium	3.81	3.53	3.7E-06
5aa6c3497cd89eac74d0549ff80cebb5	Firmicutes; Bacilli; Lactobacillales; Lactobacillaceae; Lactobacillus;	3.80	3.41	7.4E-06
e127450ed2ad1088a651c6a3f8a405b3	Bacteroidota; Bacteroidia; Bacteroidales; Marinifilaceae; Odoribacter; uncultured_bacterium	3.67	3.25	5.4E-12
bf7348b2028d96bee8686238f2bc5aa2	Bacteroidota; Bacteroidia; Bacteroidales; Rikenellaceae; Rikenellaceae_RC9_gut_group; uncultured_bacterium	3.67	3.19	1.5E-14
940c5c7f5d291961c87dd25da8580e29	Firmicutes; Bacilli; Erysipelotrichales; Erysipelotrichaceae; Faecalibaculum; uncultured_bacterium	3.56	3.26	1.3E-05
3214a07beaddfd84f1a645398e7cd97c	Firmicutes; Bacilli; Lactobacillales; Lactobacillaceae; Lactobacillus;	3.59	3.17	5.2E-03
84135a0b2ffd4bf0902d223d4ee001dc	Bacteroidota; Bacteroidia; Bacteroidales; Rikenellaceae; Rikenella; uncultured_bacterium	3.54	3.00	1.3E-06
2ef1e51ab1cf99a3c6417b05a060830e	Firmicutes; Bacilli; Lactobacillales; Lactobacillaceae; Lactobacillus;	3.59	3.20	1.1E-12
fe9a3e2c5c45bec637506a2ae1eb243c	Firmicutes; Clostridia; Clostridia_vadinBB60_group; Clostridia_vadinBB60_group; Clostridia_vadinBB60_group; unidentified	3.80	3.51	2.7E-04
234166274fbb22e38900a26eb84ece58	Bacteroidota; Bacteroidia; Bacteroidales; Muribaculaceae; Muribaculaceae; uncultured_bacterium	3.38	2.79	4.3E-03
ce0c98486c0934c2f5922589f4883a46	Bacteroidota; Bacteroidia; Bacteroidales; Rikenellaceae; Alistipes; uncultured_bacterium	3.33	2.80	9.5E-03
eb1d7f519ae360e6e68a2f1520785e5e	Bacteroidota; Bacteroidia; Bacteroidales; Rikenellaceae; Alistipes; Alistipes_sp.	3.17	2.61	2.4E-06
5df1ab20c1f2be8f98a438c5f140f06c	Bacteroidota; Bacteroidia; Bacteroidales; Rikenellaceae; ;	3.13	2.84	1.8E-04
7a6a394fe7285b50ee54b56f316a3944	Bacteroidota; Bacteroidia; Bacteroidales; Rikenellaceae; Alistipes;	3.13	2.35	2.2E-03
071adc1f6119b36a43480ff85d58b23a	Bacteroidota; Bacteroidia; Bacteroidales; Rikenellaceae; Alistipes; Alistipes_obesi	2.99	2.56	3.3E-10
325ce268c8bbe026198a5ca3dbaad5b9	Firmicutes; Clostridia; Lachnospirales; Lachnospiraceae; uncultured; Clostridium_sp.	2.93	2.60	9.5E-07
d23bbe506e4ded19e9dd017b65275bab	Firmicutes; Clostridia; Christensenellales; Christensenellaceae; uncultured; uncultured_bacterium	2.76	2.63	1.4E-13
6116fd7dc95407952dfa6a8cad835556	Firmicutes; Bacilli; Erysipelotrichales; Erysipelatoclostridiaceae; Erysipelatoclostridiaceae; uncultured_bacterium	2.67	2.45	3.0E-09
a92f4a553501103ffc108daf4c248191	Firmicutes; Clostridia; Clostridia_vadinBB60_group; Clostridia_vadinBB60_group; Clostridia_vadinBB60_group; unidentified	2.36	2.31	1.3E-04
3883c3ab093249287e47ca21ff186ca4	Firmicutes; Clostridia; Lachnospirales; Lachnospiraceae; GCA-900066575; uncultured_bacterium	2.44	2.29	1.6E-05
283f835321c660f3afd33edd88c984af	Actinobacteriota; Coriobacteriia; Coriobacteriales; Coriobacteriales_Incertae_Sedis; uncultured;	1.97	2.59	1.5E-03

Table S5: Indicator species from LEfSe analysis on microbial composition of "Late Infection" (Day 7-21 pi) mice.

OTUID.x	taxonomy	LogMax Mean	LDA	pValue
a9b24370bdb02a8ee91855de7b8b2813	Actinobacteriota; Actinobacteria; Bifidobacteriales; Bifidobacteriaceae; Bifidobacterium;	5.31	4.99	0.0E+00
129500150cad877075ed12b0c9424282	Bacteroidota; Bacteroidia; Bacteroidales; Muribaculaceae; Muribaculaceae; uncultured_bacterium	4.67	4.23	5.1E-05
d46e2205f0c6ecf67b51f83d111c509c	Proteobacteria; Gammaproteobacteria; Enterobacterales; Enterobacteriaceae; Escherichia-Shigella;	4.14	3.90	3.8E-11
7b28c20e72c6c95b3e604f0849245770	Verrucomicrobiota; Verrucomicrobiae; Verrucomicrobiales; Akkermansiaceae; Akkermansia; uncultured_bacterium	4.25	3.88	1.5E-05
153c93e302c4700f4c3515546aa47a1f	Campilobacterota; Campylobacteria; Campylobacterales; Helicobacteraceae; Helicobacter;	4.24	3.69	5.7E-03
a927fa934cf9d977992764c4dcba0b7d	Firmicutes; Clostridia; Lachnospirales; Lachnospiraceae; Lachnospiraceae_NK4A136_group;	4.46	4.14	3.3E-06
19bb6fdfb210eda549aee95a652e0fdf	Bacteroidota; Bacteroidia; Bacteroidales; Muribaculaceae; Muribaculaceae;	3.92	3.31	3.0E-03
b7aa21bce8178648a994aa472efa61eb	Bacteroidota; Bacteroidia; Bacteroidales; Marinifilaceae; Odoribacter; uncultured_bacterium	3.87	3.43	8.5E-11
878fc53ff6f6333a92673b700951fa1b	Bacteroidota; Bacteroidia; Bacteroidales; Tannerellaceae; Parabacteroides; Parabacteroides_distasonis	3.46	2.97	1.8E-02
759456dbbcad59cfb49ade008456f99e	Bacteroidota; Bacteroidia; Bacteroidales; Muribaculaceae; Muribaculaceae; uncultured_bacterium	3.62	3.24	3.7E-07
6ae0f1bc3a85b359e48647caca3cc071	Bacteroidota; Bacteroidia; Bacteroidales; Muribaculaceae; Muribaculaceae; uncultured_bacterium	3.30	2.97	4.8E-08
aae626812acc320e71e44fd7ab0561b0	Bacteroidota; Bacteroidia; Bacteroidales; Muribaculaceae; Muribaculaceae; uncultured_bacterium	3.31	3.00	5.9E-10
e1d34bfaee639f5be81d28b8b33c5e2f	Firmicutes; Clostridia; Lachnospirales; Lachnospiraceae;	2.57	2.35	9.3E-05

Table S6. qPCR Primers used in this study

Target:	Forward (5' → 3')	Reverse (5' → 3')
**cldn1**	GATGTGGATGGCTGTCATTG	CCTGGCCAAATTCATACCTG
**cramp**	CCCAAGTCTGTGAGGTTCCG	AGGCAGGCCTACTACTCTGG
**defcr6**	CAGGCTGTGTCTGTCTCTTTTG	TAAATGACCCTTTCTGCAGGTC
**16S**	GGTGAATACGTTCCCGG	CCTGTGAAGCGTCACTGTGT
**GAPDH**	TGCACCACCAACTGCTTAGC	GGCATGGACTGTGGTCATGAG
**GPR109a**	ATGGCGAGGCATATCTGTGTAGCA	TCCTGCCTGAGCAGAACAAGATGA
**GPR41**	TCCTGCCGTTTCGCATGGTGG	ACCGCCGTCAGGAAGAGGGAG
**GPR43**	AATTTCCTGGTGTGCTTTGG	ACCAGACCAACTTCTGGGTG
**IL-22**	CAACTTCCAGCAGCCATACA	GTTGAGCACCTGCTTCATCA
**muc1**	TACCCTACCTACCACACTCACG	CTGCTACTGCCATTACCTGC
**muc2**	CACCAACACGTCAAAAATCG	GGTCTCTCGATCACCACCAT
**muc3**	CTTCCAGCCTTCCCTAAACC	TCCACAGATCCATGCAAAAC
**muc4**	GAGAGTTCCCTGGCTGTGTC	GGACATGGGTGTCTGTGTTG
**pIgR**	TCAGTCCAGCACCATAGCTG	GAACGCCTCACACTCTCTCC
**reg3β**	CTGCCTTAGACCGTGCTTTC	CCCTTGTCCATGATGCTCTT
**reg3γ**	TTCCTGTCCTCCATGATCAAAA	CATCCACCTCTGTTGGGTTCA
**relmβ**	AGCTCTCAGTCGTCAAGAGCCTAA	CACAAGCACATCCAGTGACAACCA
**TJP1**	ACCCGAAACTGATGCTGTGGATAG	AAATGGCCGGGCAGAACTTGTGTA
**TLR2**	CATCACCGGTCAGAAAACAA	GTCACCATGGCCAATGTAGG
**TLR4**	TTTATTCAGAGCCGTTGGTG	CAGAGGATTGTCCTCCCATT
**β-defensin14**	GTATTCCTCATCTTGTTCTTGG	AAGTACAGCACACCGGCCAC
